# Adaptive monitoring in action—what drives arthropod diversity and composition in central European beech forests?

**DOI:** 10.1007/s10661-024-12592-4

**Published:** 2024-04-24

**Authors:** Constanze Keye, Marcus Schmidt, Christian Roschak, Wolfgang H. O. Dorow, Viktor Hartung, Steffen U. Pauls, Alexander Schneider, Christian Ammer, Laura Zeller, Peter Meyer

**Affiliations:** 1 Department for Forest Nature Conservation, Northwest German Forest Research Institute, Prof.-Oelkers-Str. 6, 34346 Hann. Münden, Germany; 2 Senckenberg Research Institute and Natural History Museum Frankfurt, Senckenberganlage 25, 60325 Frankfurt Am Main, Germany; 3 LWL-Museum of Natural History - Westphalian State Museum with Planetarium, Sentruper Str. 285, 48161 Münster, Germany; 4 Institute of Insect Biotechnology, Justus-Liebig-University, Heinrich-Buff-Ring 26-32, 35392 Gießen, Germany; 5 Department of Silviculture and Forest Ecology of the Temperate Zones, University of Göttingen, Büsgenweg 1, 37077 Göttingen, Germany; 6 New Zealand Forest Research Institute Ltd (Scion), Te Papa Tipu Innovation Park Tītokorangi Drive, 3020 Rotorua, New Zealand

**Keywords:** Species richness, Species composition, Monitoring, Indicator selection, Forest structure

## Abstract

Recent studies suggest that arthropod diversity in German forests is declining. Currently, different national programs are being developed to monitor arthropod trends and to unravel the effects of forest management on biodiversity in forests. To establish effective long-term monitoring programs, a set of drivers of arthropod diversity and composition as well as suitable species groups have to be identified. To aid in answering these questions, we investigated arthropod data collected in four Hessian forest reserves (FR) in the 1990s. To fully utilize this data set, we combined it with results from a retrospective structural sampling design applied at the original trap locations in central European beech (*Fagus sylvatica*) forests. As expected, the importance of the different forest structural, vegetation, and site attributes differed largely between the investigated arthropod groups: beetles, spiders, Aculeata, and true bugs. Measures related to light availability and temperature such as canopy cover or potential radiation were important to all groups affecting either richness, composition, or both. Spiders and true bugs were affected by the broadest range of explanatory variables, which makes them a good choice for monitoring general trends. For targeted monitoring focused on forestry-related effects on biodiversity, rove and ground beetles seem more suitable. Both groups were driven by a narrower, more management-related set of variables. Most importantly, our study approach shows that it is possible to utilize older biodiversity survey data. Although, in our case, there are strong restrictions due to the long time between species and structural attribute sampling.

**Supplementary Information:**

The online version contains supplementary material available at 10.1007/s10661-024-12592-4.

## Introduction

Recent evidence suggests that arthropod abundance and diversity are declining both locally (Hallmann et al., 2017; Seibold et al., 2019) and globally (Wagner, 2020). The reasons are complex and not entirely understood. Habitat degradation and loss through management actions at local and especially at landscape scale, as well as changes in climatic conditions are suspected (Seibold et al., 2019; Uhler et al., 2021). Most central European forests are managed for timber production and other ecosystem services. Under the paradigm of multifunctionality which is widely applied in public forests, the preservation of biodiversity is a central goal of forest management (Borrass et al., 2017). Therefore, to react appropriately to the diversity decline, the underlying causes must be better understood. In particular, the role of climate change and management actions on biodiversity needs to be unraveled further, especially in forest ecosystems (Ammer et al., 2018). The vast majority of research on relationships between forest structure and biodiversity focused on the stand level (e.g. J. Müller et al., 2008a, 2008b; Paillet et al., 2010; Schauer et al., 2018), with notable exceptions in Germany (e.g. Fischer et al., 2010; Schall et al., 2018). To investigate arthropod trends and possible causes for the decline, new large-scale long-term monitoring concepts are currently being developed in Germany (Hagge et al., 2021). However, it will take time until these new programs generate results. One way to bridge this period and simultaneously aid the development of these programs is to fully utilize the potential of existing data pools (Kindsvater et al., 2018). It can be a difficult question whether it is reasonable to continue established sampling protocols. Many older biodiversity monitoring programs are in some ways flawed in their data collection or processing procedure (Archaux, 2011). On the other hand, especially long-standing time-series are both essential (Likens, 1989; F. Müller et al., 2010) and still scarce (Meyer, 2020).

We here demonstrate how existing data sets can be utilized using the Hessian forest reserves program as an example. This forest biodiversity monitoring program has been ongoing since 1988 in the federal state of Hesse, Germany (Schneider et al., 2021a, 2021b). Data have been collected in permanently designated pairs of stands consisting of a strict forest reserve (SFR), where forestry interventions ceased at the latest in the year of designation (most SFR were designated in 1988), and an adjacent managed reference area (MRA) (Blick et al., 2012). Development of the tree stands and their structure was monitored by the responsible forest research institutes (since 2006: Northwest German Forest Research Institute = NW-FVA), while the Senckenberg Society for Nature Research (SGN) collected data on six invertebrate groups (Heteroptera, Aculeata, Araneae, Coleoptera, Macrolepidoptera, and Lumbricidae) intermittently since 1990. Arthropod traps were placed independently from the grid-based NW-FVA forest structural plots to maximize the diversity of the sampled habitats. Although a protocol (SGN) for assessing structural attributes at trap locations existed, it was not well-defined and changed over time. Soon it was obvious that these different sampling designs impeded the joined analysis of both (NW-FVA and SGN) data sets, whereas the original trap structural protocol was too unstructured to be of much help. Shortcomings such as these are common for long-standing monitoring schemes (Lindenmayer & Likens, 2018; Meyer, 2020). Consequently, since 2006, both organizations have focused on consolidating and complementing the initiative, with one important aim being to allow a causal analysis of the arthropod data set and forest structural information.

**Fig. 1:**
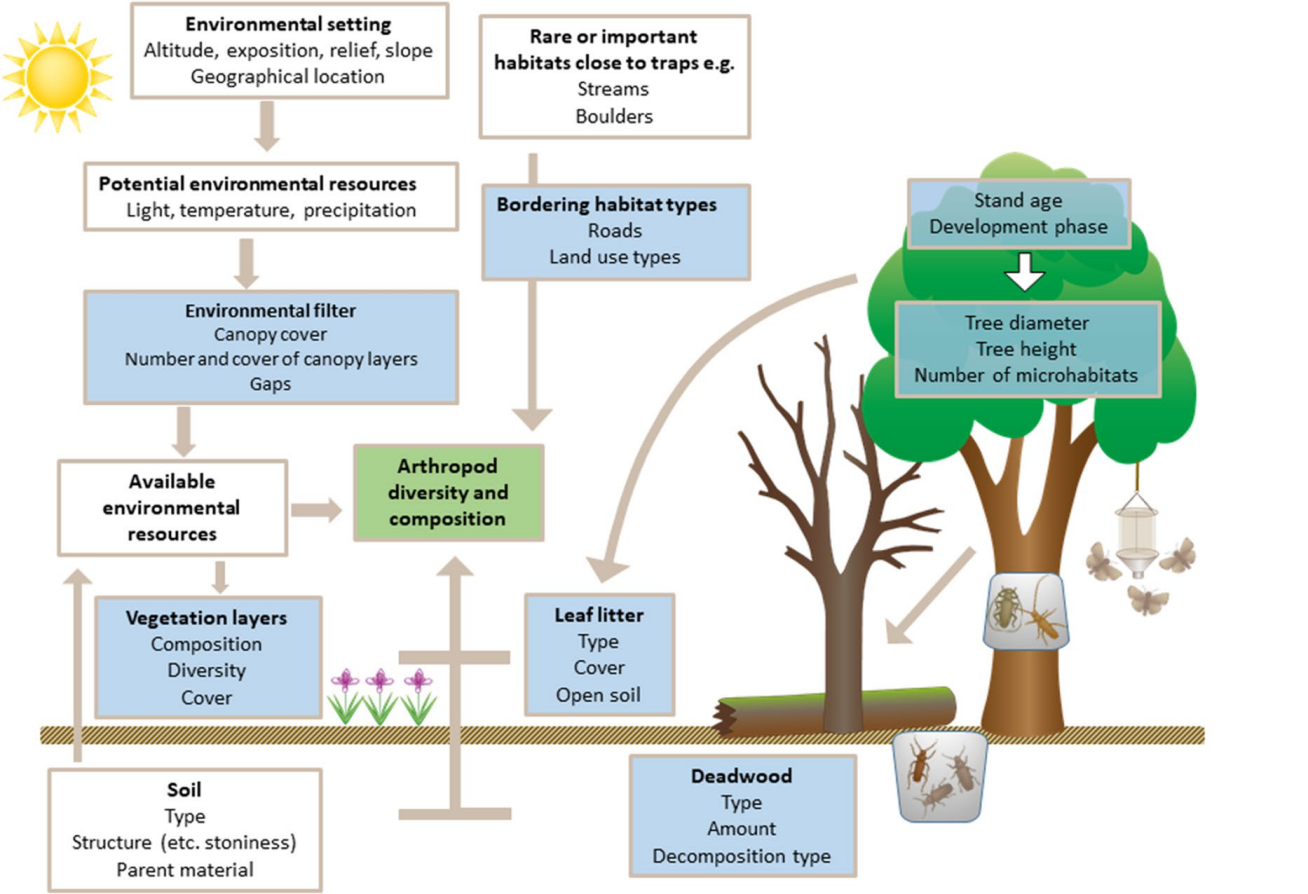
Relationship between environmental, vegetation structural and compositional variables, natural habitats or management-related habitats, and arthropod composition and diversity. Variables that can directly or indirectly be influenced by management actions are marked in blue. The three different trap types used for the data analysis of this paper are shown (pitfall traps, flight interceptor traps, and eclector traps)

As most of the faunistic sampling was conducted in the first half of the 1990s, a robust method had to be designed that was suitable to retrieve structural data retrospectively. Forest structures important for arthropods were identified based on a conceptual model as suggested by Lindenmayer and Likens (2009) (Fig. 1). Our study assessed all of these variables, except for the potential environmental resources group.

Subsequently, both institutions developed a sampling protocol for recording the identified variables retrospectively in 2018 at the former trap locations. Retrospectively means the assessment of the condition of the structural variables at the time of arthropod sampling. The original trap structural protocol from the time of arthropod sampling was used together with aerial pictures from the 90 s to verify the sampled structural variables.

Using the old arthropod data set and the newly gained information on structural attributes, it was possible to investigate some causal questions to aid the development of the new national arthropod monitoring programs in Germany.

First of all, we needed to evaluate if our retrospective approach led to meaningful results, given the relatively long time span between arthropod sampling and assessment of the environment. In case our approach was successful, we wanted to address the following questions: (1) which variables drive arthropod communities in beech forests, (2) are species richness and community composition driven by the same variables, (3) is species richness correlated between species groups, and (4) which arthropod groups may be suitable for a general trend or targeted monitoring (i.e., effect of forest management)? For the latter, we used an indirect approach by classifying structural variables into those that can or cannot be affected by forest management.

## Material and methods

### Study region

**Fig. 2:**
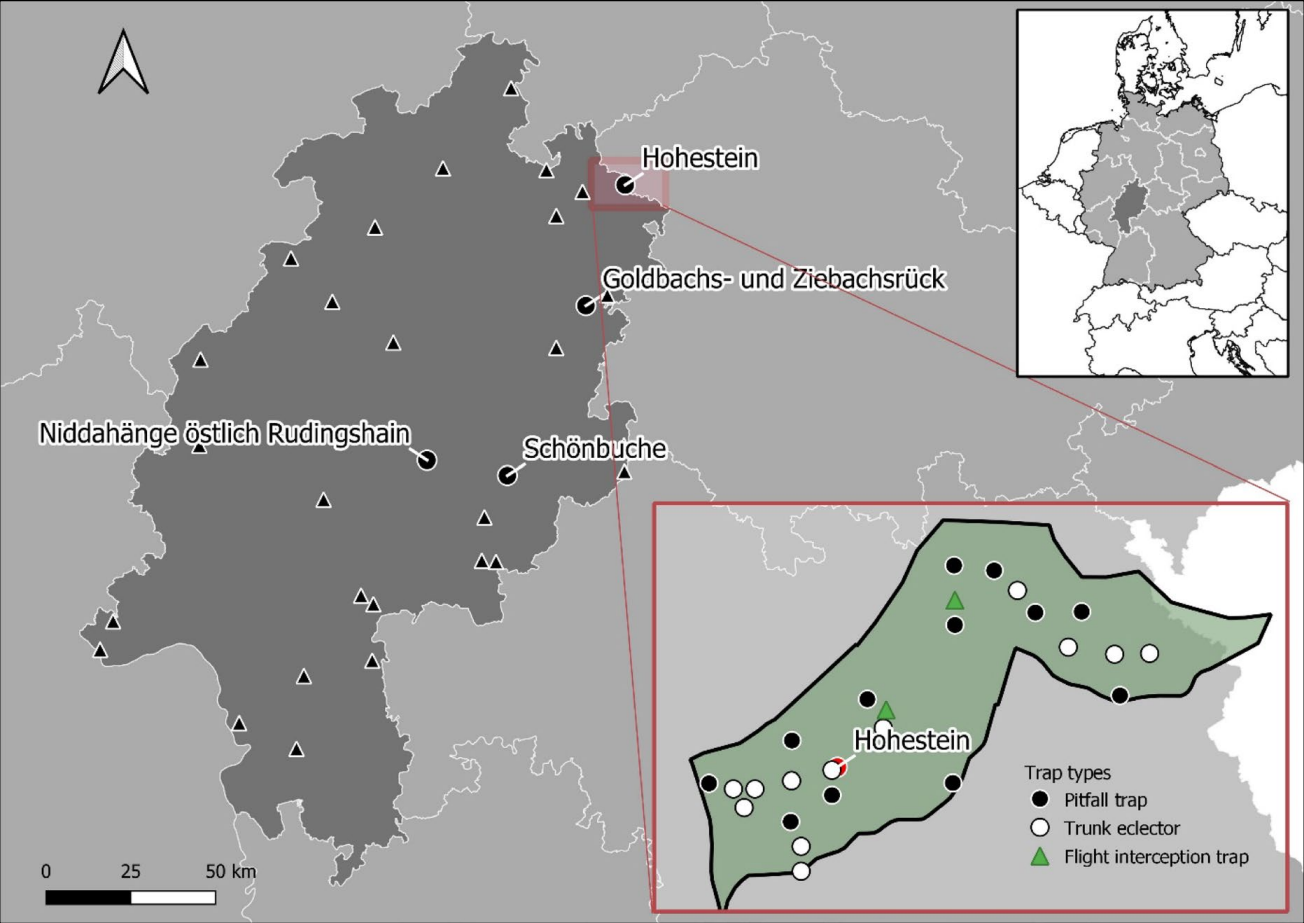
Location of all 31 strict forest reserves (triangles) including the four study sites (dots) in the federal state of Hessen in Germany (WGS84 coordinate system). The forest reserve “Hohestein” was used as an example to show the arthropod trap setup at the study sites. Note, that these traps are at the same time the center of the forest structural plots recorded, retrospectively

This study was conducted in the federal state of Hesse in the southwest of Germany (Fig. 2). A total of 31 forest reserves (FR) were designated since 1988. This study is based on data collected in four of these reserves and associated managed sites (Table 1). These four study areas were the first for which an extensive arthropod data set was collected and analyzed at species level. Management ceased on these FR sites in 1988. They are dominated by natural beech communities (*Fagus sylvatica*) that are generally in the “optimal” phase of stand development, i.e., fully stocked mature beech stands with low mortality. Reserve areas range from 51 to 74 ha. The continuously managed reference areas (MRA) are directly adjacent to the SFR. Parent material in two of the reserves is red sandstone, while the other two are based on shelly limestone and basalt.

**Table 1:** Main environmental, geographic, ecological, and arthropod trap information of the four study areas. Values for the whole forest reserves (FR) are given here. Managed (MRA) and unmanaged (SFR) forest reserve sites were very similar, as management ceased only a few years before arthropod sampling at the SFR establishment date. For more information, see Blick et al., (2007, 2009, 2010, 2011), Dorow et al. (2004), and Schreiber et al. (1999)

Study area	Goldbachs-und Ziebachsrück	Hohestein	Niddahänge östlich Rudingshain	Schönbuche
Arthropod sampling period	1994–1996	1994–1996	1990–1992	1990–1992
Main potential natural vegetation type	Luzulo-Fagetum	Hordelymo-Fagetum	Galio odorati-Fagetum/Hordelymo-Fagetum	Luzulo-Fagetum
Stand age (min/max)	121–171	30–150	103–223	135–146
Area (ha)	68	51	74	55
Altitude (min/max)	300/365	455/565	530/690	370/455
Mean annual precipitation (mm year)	796	1024	1230	919
Mean annual temperature (°C)	8.4	7.2	13.4	8
Longitude	9.875014	10.046786	9.203562	9.539696
Latitude	50.930089	51.249027	50.523953	50.482362
Pitfall traps (*n*)	15	12	14	12
Trunk eclectors/flight interception traps and window traps (*n*)	12/2	12/2	20/4	12/4

### Arthropod sampling and grouping

Arthropod sampling was carried out from 1990 to 1996; however, actual sampling times differed between areas (Table 1). A stratified sampling approach was applied (more information can be found in Table S1 and S2 in Supporting information 1). Stratification was done according to different larger habitat structures associated with different successional forest stages from a zoological point of view (e.g., open spaces, bare ground or areas dominated by pioneer plant species, or different forest vegetation communities). Each of these habitat structures found in the study areas was sampled using pitfall traps. Here, ground-dwelling arthropods were caught using three of these traps (10 cm diameter with tin roofs) per sampling location, spaced 5 m apart. The understory layer was sampled with a minimum of 12 trunk eclectors (ecoTECH, Bonn) per study area, mounted at least onto two trees of the dominant tree species of the following categories: lying, standing and dead, and standing and alive. For flying arthropods, at least two window traps (WT) or flight interceptor traps (FIT) (ecoTECH, Bonn) were set up for each area. The WT and FITs were elevated 1.5 m above ground (Dorow et al., 1992). A two part ethanol (70%) and one part glycerine (86%) mixture with a small addition of a commercial detergent was used as trapping liquid. Traps were checked monthly over a period of 2 years through the growing season. Over winter, traps were left unchecked until April. Trunk eclectors were installed on living and dead standing trees and on fallen trees. Detailed information about the arthropod sampling protocol and identification keys used are given in Dorow et al., (2004, 2007, 2009) and Flechtner et al., (1999, 2000). For this study, data on true bugs (Heteroptera), Aculeata, spiders (Araneae), and beetles (Coleoptera) were analyzed. All individuals of these groups were identified to species level by entomologists given in Schneider et al., (2021a, 2021b) (Table 3). Aculeata were analyzed using the data for the understory layer; ants (Formicidae) were excluded because trap types used were not effective enough for a causal analysis (too few individuals caught).

In our data set, beetles were the most diverse group, representing a variety of functional groups. Therefore, they were further divided into the most abundant and species-rich groups sampled in pitfall traps (ground-dwelling beetles): ground beetles (Carabidae) and rove beetles (Staphylinidae). We also classified the ground-dwelling beetles into species associated with forests (forest affinity) following Dorow et al. (2019), as these might be more sensitive to management-related habitat changes (Fuller et al., 2008; Lange et al., 2014). The following forest affinity classes were included in this group, as described by Schneider et al., (2021a, 2021b): (f) mainly found in forests without preference for light or closed forests, (fc) mainly found in forests with strong affinity to closed forest habitats, and (fl) mainly found in forests with strong affinity to light forests, forest edges, or glades. Beetles caught in the understory stratum were grouped into saproxylic species which depend on deadwood during at least part of their life cycle (Speight, 1989) and non-saproxylic beetles. For assigning beetles to the saproxylic community, Stokland et al. (2012) and the German reference list of saproxylic beetles (Köhler, 2000; Schmidl & Bussler, 2004) were used.

### Vegetation structure and habitat sampling

Over 130 stand attributes were recorded belonging to six main groups in 2018: site characteristics, soil variables, forest structure, vegetation, deadwood, microhabitats on eclector trap trees, and important habitat types (see Supporting Information 2 for more information). Important habitat types were defined as being either nationally red-listed (Finck et al., 2017) or of potential importance to arthropod communities, e.g., deep wheel tracks temporarily filled with water or disturbance-related habitats such as paved or unpaved roads. Stand attributes were assessed using concentric circular sampling plots, with the trap as the plot center. If several trap types were installed at the same site, one was used as the plot center, and structural attributes were assessed only once for all traps. Most of the attributes were sampled in a 10-m radius around the trap locations. Variables related to stand properties of larger spatial extent such as canopy gaps and important habitats were assessed in a 30-m radius circular plot. Table 2 provides details for the variables used in advanced analyses (RDA, regression boosting) of this study. For information about the other variables, see supplement 2. Note that the retrospective assessment of stand variables was done over 20 years after arthropod sampling. Every possible effort was made to estimate the attributes recorded at their condition at the time of arthropod sampling. Field notes on structural attributes recorded during the time of trapping activity and aerial pictures (light aircraft) from the years of arthropod sampling were used. Some attributes, such as coarse woody debris and snags, were only recorded with presence/absence. Therefore, this results in strong limitation for this study. For example, the existence of microhabitats on eclector trees at time of sampling was hard to assess after that time span. Therefore, the accuracy of this retrospective approach differs between attributes.

**Table 2:** Giving a short description of the forest structural and habitat variables used for the final analyses. Descriptive statistics are given for the sampling plots associated with pitfall traps. Percentage cover was recorded visually according to modified Braun-Blanquet (1932) cover class ranges: 0 = absent, 1 < 5%, 5 = 5– < 25%, 25 = 26– < 50%, 50 = 51– < 75%, and 75 >  = 75%) for all variables were % class (unit) is given, except for stoniness

Variable	Description	Unit	Plot radius	Mean ± SE	Observed range
Stand structure					
Canopy cover	Estimated % cover of the highest tree layer	% class	10 m	64.5 ± 1.9	0–75
Shrub cover	% cover of woody plants > 0.5 m and dbh (diameter at breast height) < 7 cm	% class	10 m	9.9 ± 1.1	0–75
Snags	Standing broadleaf or coniferous deadwood > 7 cm dbh in different sizes (7–20, 20–50, > 50 cm dbh) and three decomposition classes. Values were only recorded with presence and absence. The occurrence of deadwood was aggregated at plot level over all classes (e.g., deadwood present in three classes and only one decomposition class, score 3). The value therefore indicates snag diversity rather than volume	Index	10 m	0.8 ± 0.1	0–5
CWD	Lying broadleaf or coniferous deadwood > 20 cm diameter over different size classes (20–50, > 50 cm diameter). Presence and absence of deadwood, differentiated in three decomposition classes was estimated. The occurrence of deadwood was aggregated at plot level over all classes and therefore indicates CWD diversity rather than volume (see example in snags)	Index	10 m	1.2 ± 0.1	0–6
Stumps	All broadleaf stumps ( conifers were very scarce in the study areas)	Count	10 m	2.3 ± 0.2	0–15
Vegetation diversity and composition					
Tree richness	Number of tree species in the upper most canopy layer. The 5 most dominant tree species were recorded based on their % cover estimate	Count	10 m	2.4 ± 0.1	0–5
Oak cover	Estimated % cover of oak species in the highest tree layer	% class	10 m	0.6 ± 0.7	0–25
Herbs (plant groups)	Most dominant herbaceous or woody species group within the ground tier of 0–0.5 m height (five types: herbaceous, ferns, Poaceae, Cyperaceae, dwarf shrubs, and shrubs)		10 m	-	5
Site variables					
Radiation	Calculated direct solar open beam radiation for a given day of year, location and topography (function = DirectRadiation, package solrad)	W/m^2^	calc	671 ± 92.3	254–820
Leaf litter depth	Estimated thickness of the humus layer	cm	10 m	2.5 ± 0.1	0–7
Bare soil	Cover estimate of open mineral soil without any leaf litter	% class	10 m	1.7 ± 0.1	0–25
Stoniness	Estimated volume proportion of stones in topsoil (0–10 cm depth). Percentage classes used were 0 = 0%, 1 = < 2%, 2 = 2%– < 10%, 3 = 10%– < 25%, 4 = 25%– < 50%, 5 = 50%– < 75%, 6 = ≥ 75%	% class	10 m	1.3 ± 0.1	0–5
Rocks	Cover % of larger rock and stone habitats (cluster of stones with > 200 mm diameter, rocks, boulders, or scree)	% class	10 m	5.5 ± 0.6	0–80
Vertical structures					
Gaps	A large canopy gap which has to be closed by regenerating trees rather than by neighboring trees	y/n	30 m	-	-
Roads	Unpaved road/path dominated by grass	% class	30 m	9.4 ± 0.5	0–50
Co-variable					
FR	Identity of the Strict Forest Reserve/including the continuously managed comparison sites	-	-	-	4

### Data analysis

#### Data exploration and preparation

In our data analysis, we did not distinguish between SFR and MRA, as at the time of sampling, management ceased only for a couple of years in the FR. The two adjacent areas are hereinafter referred to as forest reserves (FR). Species abundance data was reduced to presence–absence, as sampling years differed in forest reserves, which might affect the numbers of individuals caught and the species numbers encountered. We addressed this issue by inspecting sample completeness curves and subsequently equalizing the sample completeness (see section: “Univariate analysis of species richness”). Field seasons were standardized to include only June–November catches as sampling intervals and duration varied for the remaining months between areas. Species were aggregated over the two sampling years, by FR and trap, as we were not interested in the effect of seasonality. The forest structural data set was used as explanatory data (*n* = 130), and the arthropod richness and composition of the different groups was set to be the response. Data exploration following the protocol suggested by Zuur et al. (2010) was carried out to prevent type I and type II errors in our analysis. Variables that correlated with FR identity (e.g., altitude), were removed, as FR was used as covariate or constraint during analysis. Explanatory variables, e.g., rare habitats that were encountered just once in the field were removed. All analyses were performed using R statistical software (v. 4.2.1, R Core Team, 2022). Correlation between explanatory variables was checked using functions “pairs” and “cor,” and highly correlated explanatory variables were removed. Some variables were aggregated such as deadwood. Snags and coarse woody debris (CWD) were only assessed with presence/absence, because of the retrospective nature of the sampling. To simplify the analysis, we combined for each type (e.g., CWD) these occurrences at the plot level over all diameter and decomposition classes (see example in Table 2). Deadwood values used in this analysis consequently indicate snag/CWD diversity rather than volume or count.

To further reduce the number of explanatory variables (*n* = 25) in the data set, backward stepwise variable selection using consecutive principal component analyses (PCA) was performed using function PCAmix from the PCAmixdata package (Chavent et al., 2017). A process explained in detail by King and Jackson (1999) is named “B1Backward.” The last principal component with eigenvalues > 0.70 (Jolliffe, 1973) of the initial PCA was examined, and the variable with the highest loading was removed. Subsequent PCAs were carried out, repeating this process until all axes have eigenvalues > 0.70. The resulting set of variables (*n* = 15) can be seen in Table 2.

To assess the loss of information in the first and the final PCA, we performed a symmetric Procrustes analysis (Peres-Neto & Jackson, 2001) using function protest from the vegan package (Oksanen et al., 2022). The two PCA results were significantly correlated and the sum of squares of the symmetric analysis (m^2^_12_; 0 < m^2^_12_ < 1_)_ was relatively low, indicating a good fit between both ordinations (correlation in a symmetric Procrustes rotation = 0.77, *p* = 0.001, m^2^_12_ = 0.39).

Transformation of structural variables was not necessary as no extreme observations were found.

### Multivariate analysis of species composition

Rare species, i.e., species which occurred in less than two samples, were removed from the species data set to reduce noise in the subsequent analysis (McCune & Grace, 2002). Species data were Hellinger transformed to avoid problems associated with Euclidean distances such as the effect of double-zeros as suggested by Legendre and Gallagher (2001). Importance of structural variables to species composition was determined using a forward selection procedure applying a partial transformation-based redundancy analysis (tb-RDA). We followed the protocol by Blanchet et al. (2008) to avoid common issues caused by stepwise variable selection, overestimating the amount of explained variance and a highly inflated type I error. At first, a global partial tb-RDA using function RDA (vegan package) including all explanatory variables was carried out. Analysis only proceeded if the overall model was significant. The variance inflation factor (VIF) for each variable was inspected to examine multicollinearity between variables. All variables with VIF values > 5 were checked and if necessary excluded from further analysis.

Second, function “ordi2step” from the vegan package was used for forward selection of structural variables. We applied the two stopping criteria recommended by Blanchet et al. (2008): the function stopped if the adjusted *R*^2^ (e.g., eigenvalues) of the new model was exceeding the adjusted *R*^2^ of the global model (using argument R2scope). The second criterion was the significance of the new variables’ additional contribution to the model accessed (default setting: Pin = 0.05). The “best” variable in each iteration is the variable that explains the largest portion of the remaining variation (Legendre & Legendre, 2012). The effect of the individual strict forest reserves was partialled out. Significance of variance explained by each variable was determined by a Monte–Carlo permutation test (*n* = 9999) by terms (each term sequentially tested), and permutation was restricted to be calculated only within each reserve. *p* values were corrected for multiple testing using Benjamini and Hochberg (1995) correction. Uncorrected and corrected *p* values are shown in the result section, as we were mainly interested in the order of importance of variables, not in absolute differences. Zuur et al. (2007) did not correct *p* values in their order of importance analysis and suggested interpreting results carefully, especially ones that are close to the significance level.

### Univariate analysis of species richness

Species richness for each species group was rarefied or extrapolated using function estimateD from package iNEXT (Hsieh et al., 2022) on abundance data. Sample completeness was equalized to double the observed sample size for each species group as recommended by Chao and Jost (2012). Sample completeness is a measure of how completely a community has been sampled. The information loss in comparison to rarefaction methods where samples are standardized to the lowest sample size is small. A more complete picture of the community can be retained; hence, sample completeness estimators have been suggested to be the preferred method to equalize samples (Roswell et al., 2021).

We applied a component-wise gradient boosting algorithm, using the function gamboost from the package “mboost” (Hothorn et al., 2022), to model the effect of structural parameters on species richness. This machine-learning approach avoids problems such as overfitting as the model is built in a sequential manner (Mayr et al., 2017). Gradient boosting is therefore especially suitable for data sets with large numbers of collinearity (Hothorn et al., 2011). Unlike in stepwise variable selection, variable selection is carried out during the fitting process (Bühlmann, 2006). We used a gamma distribution for the loss function (response) and linear models for the reduced set of explanatory variables (baselearners). A 15-fold (bootstrap) cross-validation was used to select the optimal model (mstop) and prevent overfitting. Variable importance was calculated from the individual contribution to risk reduction of each baselearner up to the optimal iteration number (mstop) using function “varimp.”

In some cases, pitfall traps, eclector traps, and window traps or panes were set up at different locations, being more than 50 m apart. To inspect the relationship of species richness between groups, we therefore averaged richness for the two nearest pitfall trap locations and used the result for the understory trap types. Package “FNN” (Beygelzimer et al., 2019) was applied to identify the *k*-nearest pitfall trap neighbor for each understory trap type. Spearman’s rank partial correlation coefficient was calculated using “partial.r” from package psych (Revelle, 2021), to partial out the effect of the Strict Forest Reserves. Associated *p* values and correlation plots were constructed with “corrplot” from package “corrplot” (Wei & Simko, 2021).

## Results

### Which variables drive species composition?

Our set of explanatory variables had a significant effect on species composition for all groups (global model; *p* ≤ 0.05, Table 3), except for beetles associated with forests (forest specialists). The effect of the forest reserve identity on species composition differed greatly between groups, ranging from 14% explained variance for saproxylic beetles up to 37% for ground beetles. The explanatory power of the structural and habitat variable set was less diverse ranging from 27% of explained variance for ground beetles to 37% for rove beetles. A large number of variation in the data set was left unexplained (36–55%). The highest proportion of unexplained variance, 55%, was found for the group of saproxylic beetles in the understory tier. Effect size (Pseudo *F* 1.1–2.11, perm 49,999) of single habitat variables was small for all species groups.

**Table 3:** General data set information such as species that appeared only once in the data (singletons) and number of habitat variables tested. Results of global RDA models including all structural variables which were significant at the level of 0.05 are presented. The total percentage of variance explained by strict forest reserves (conditional), habitat variables (constrained), and unexplained variance (unconstrained) are given. Species groups were sampled in different tiers: U, understory: tree trunks and air; G, ground. The number of sampling plots used for community and species richness analyses is indicated under “Plots.”

					Variance (total, %)			
**Species group**	**Tier**	**Species**	**Singletons**	**Plots**	**Total**	**Cond**	**Const**	**Unconstr**
Beetles	G	640	214	53	0.62	0.13 (21)	0.22 (35)	0.27 (43)
Rove beetles	G	261	68	53	0.60	0.11 (19)	0.22 (37)	0.27 (44)
Ground beetles	G	73	20	53	0.57	0.20 (37)	0.16 (27)	0.20 (36)
Beetles assoc. with forests	G	298	93	53	RDA global model not significant			
Spiders	G	115	60	53	0.64	0.12 (19)	0.20 (31)	0.3 (50)
Beetles	U	897	269	66	0.72	0.09 (13)	0.23 (31)	0.40 (56)
Saproxylic beetles	U	295	67	66	0.71	0.11 (14)	0.22 (31)	0.39 (55)
Non-saproxylic beetles	U	602	202	66	0.72	0.08 (12)	0.23 (32)	0.41 (57)
True bugs	U	126	43	62	0.70	0.09 (13)	0.25 (36)	0.36 (51)
Aculeata	U	165	68	62	0.73	0.06 (08)	0.27 (37)	0.40 (54)

The order of variable importance varied widely between species groups (Table 4). No effects on any of the species groups were found for roads, snags, coarse woody debris (CWD), rocks, and bare soil. From the tested soil variables, soil stoniness was found to affect a range of arthropod groups (Table 4). Leaf litter depth was a strong determinant of ground beetle community structure (Pseudo *F* 2.167, adj. *p* ≤ 0.03). The variable “the most dominant plant group of the herbaceous layer,” e.g., herbs or ferns was for saproxylic beetles, true bugs, Aculeata, spiders, and understory beetles, the most important driver of compositional structure (Table 4). Which plant group (see Table 2 for plant groups used) had the strongest effect on arthropod assemblages differed widely (Figure S3 in Supporting information 1). RDA triplots showed that the different plant groups were also correlated with other variables, especially once indicating different light regimes represented by variables such as canopy cover and radiation (Figure S3).

**Table 4:** Explanatory variables in order of importance for arthropod composition as indicated by the RDA forward selection process. Variables that were selected by forward selection, but after *p* value correction was insignificant, are included, as we were mainly interested in the relative order of importance. Results of the permutation tests (marginal effects, perm = 49,999) for each attribute of each arthropod group are reported here

Species group	Attribute	Variance	Pseudo *F*	*p* value	Adj. *p* value (BH)
Beetles (G)	Canopy cover	0.016	1.572	0.001**	0.030*
	Herbs	0.062	1.073	0.050*	0.030*
Rove beetles (G)	Canopy cover	0.017	1.746	0.001**	0.017*
	Stumps	0.014	1.460	0.003**	0.043*
Ground beetles (G)	Leaf litter depth	0.015	2.167	0.003**	0.032*
	Tree richness	0.011	1.573	0.034*	0.272
Spiders (G)	Herbs	0.071	1.179	0.016*	0.080*
	Stoniness	0.016	1.567	0.004**	0.060*
	Canopy cover	0.015	1.503	0.009**	0.067
	Radiation	0.014	1.372	0.037*	0.138
Saproxylic beetles (U)	Herbs	0.068	1.183	0.002**	0.030*
	Stoniness	0.014	1.495	0.003**	0.030*
	Canopy cover	0.014	1.446	0.005**	0.045*
	Oak cover	0.012	1.296	0.014*	0.060
Non-saproxylic beetles (U)	Herbs	0.073	1.233	0.006**	0.060
	Stoniness	0.015	1.486	0.011*	0.060
	Canopy cover	0.015	1.433	0.013*	0.060
	Radiation	0.013	1.426	0.015*	0.060
	Shrubs	0.013	1.326	0.036*	0.106
True bugs (U)	Herbs	0.082	1.422	0.002**	0.030*
	Radiation	0.018	1.741	0.005**	0.056
	Stoniness	0.017	1.871	0.015*	0.038
	Gaps	0.017	1.808	0.013*	0.056
	Shrubs	0.016	1.685	0.021*	0.063
Aculeata (U)	Herbs	0.088	1.272	0.002**	0.040*
	Gaps	0.018	1.568	0.020*	0.155

Unexpectedly, an effect of the quantity of deadwood (stumps) was only found for rove beetles and for no other species group. From the vertical structure variable group, only gaps were found to be a driver for the true bugs and Aculeata communities. Light-related variables such as canopy cover, gaps, or radiation were important to all species groups except ground beetles. The RDA triplot (Figure S3) indicates a main gradient for the saproxylic beetle community, ranging from high canopy openness represented by the dominance of woody plants in the herbaceous layer to a closed canopy (max = 75%). Other management-related variables that had an effect on arthropod groups were oak cover (saproxylic beetles), tree richness (ground beetles), and stumps (rove beetles) (Table 4).

### Which variables drive species richness?

The effect size of most variables was at a low to medium range as depicted by the standardized model coefficients (β) of the gradient boosting models ranging from − 0.17 to 0.23. As for species composition, no effect of presence of roads in a 30-m radius and quantity of CWD on our species groups could be detected.

**Fig. 3:**
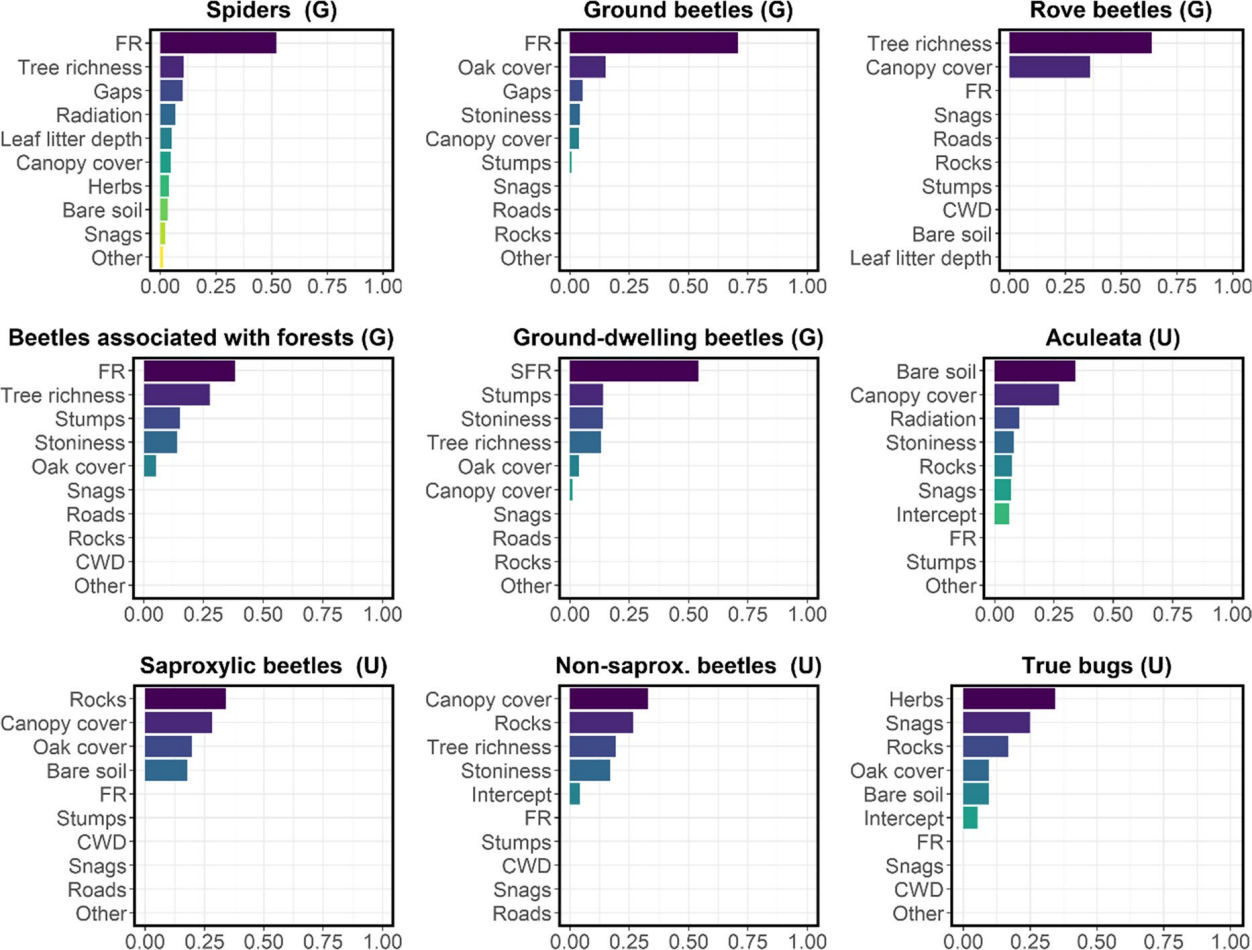
Variable importance for the prediction of (rarefied) species richness from gradient boosting models with inherent variable selection. The importance of different variables in the final model was estimated by quantifying the individual contribution to in-bag risk reduction. In-bag risk reduction was calculated as the accumulated contribution of each variable (base learner) to the final model

There was a weak to moderate effect of location (FR) on species richness for four species groups (β =  − 0.17 to 0.22). According to the in-bag risk reduction (IB risk%) for spiders (51.9%), ground beetles (70.8%), beetles associated with forests (54.1%), and ground-dwelling beetles (54.7%), the identity of the strict forest reserve was the most important variable for explaining differences in species richness (Fig. 3). Surprisingly, increasing cover % of oak trees had a negative effect on species richness in five species groups (Figs. 5 and 6). Canopy cover had a negative effect on species richness in seven of nine species groups, even though the overall effect size was rather low β <  − 0.09 (Figs. 5 and 6).

The dominant plant group on the ground floor was of low importance for spider richness (β =  − 0.004 to 0.005) in contrast to spider composition. Even though a multitude of variables drove spider richness, these all had a very minor effect (β < 0.08). The dominant plant group was the most important driver of true bugs richness (IB risk = 34.4%; Fig. 3), which corresponds with the effect for species composition.

In contrast to community composition, tree species richness had no effect on ground beetles but was the most important driver of richness for rove beetle (IB risk = 63.8%; β = 0.12) and beetles associated with forests (IB risk = 27.65.8%; β = 0.11). It also had a positive minor effect (β ≤ 0.07) on spider richness, ground-dwelling beetles, and forest beetles.

From the soil variables, leaf litter depth was only (weakly) important for spider richness (IB risk = 5.1%; β =  − 0.05). Arthropod groups reacted differently to levels of stoniness in soil. Three groups reacted positively (β ≤ 0.05) and one, the Aculeata, negatively (IB risk = 8.2%; β =  − 0.12). Both negative and positive effects on species richness were observed for cover % of bare soil in four species groups (β =  − 0.39 to 0.03). The overall effect of soil-related variables was low, with the exception of the Aculeata, which were strongly driven by the cover % of bare soil (IB risk = 34.0%; β =  − 0.39). Cover % of rocks was positively associated with true bugs (IB risk = 16.7; β = 0.16) and non-saproxylic beetle richness (IB risk = 26.6; β = 0.14). Quantity of snags had a positive but weak effect on true bugs (IB risk = 24.8; β = 0.17) and spider richness (IB risk = 2.3; β = 0.02). Interestingly, stump quantities had a weak negative effect (β ≤  − 0.06) on richness for a number of species groups (spiders, ground beetles, ground-dwelling beetles, and beetles associated with forests). It was the second most important determinant of species richness of all variables for beetles associated with forests (IB risk = 15.3; β =  − 0.07).

### Are species richness and community composition driven by the same variables?

**Fig. 4:**
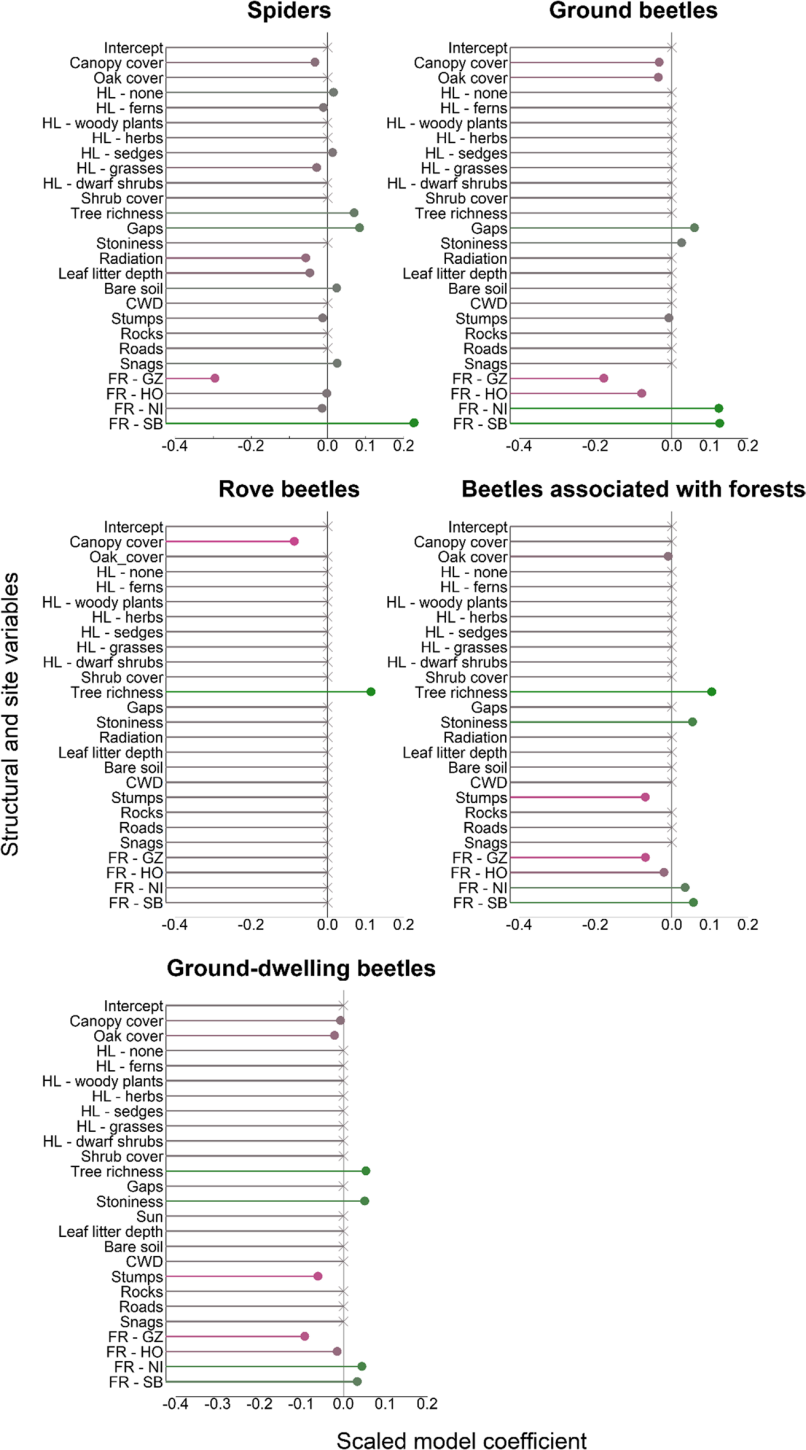
Results of the gradient boosted regression models for arthropod groups sampled on the ground layer with pitfall traps. The scaled coefficients can be interpreted as a measure of effect size. Final models were gained by tenfold cross-validation. Direction and intensity of relationships between variables and species richness are indicated by lines (point) being red (negative), or green (positive); color intensity increases with model coefficient size. Crosses depict that no or a very weak (β < 0.001) relationship was found

**Fig. 5:**
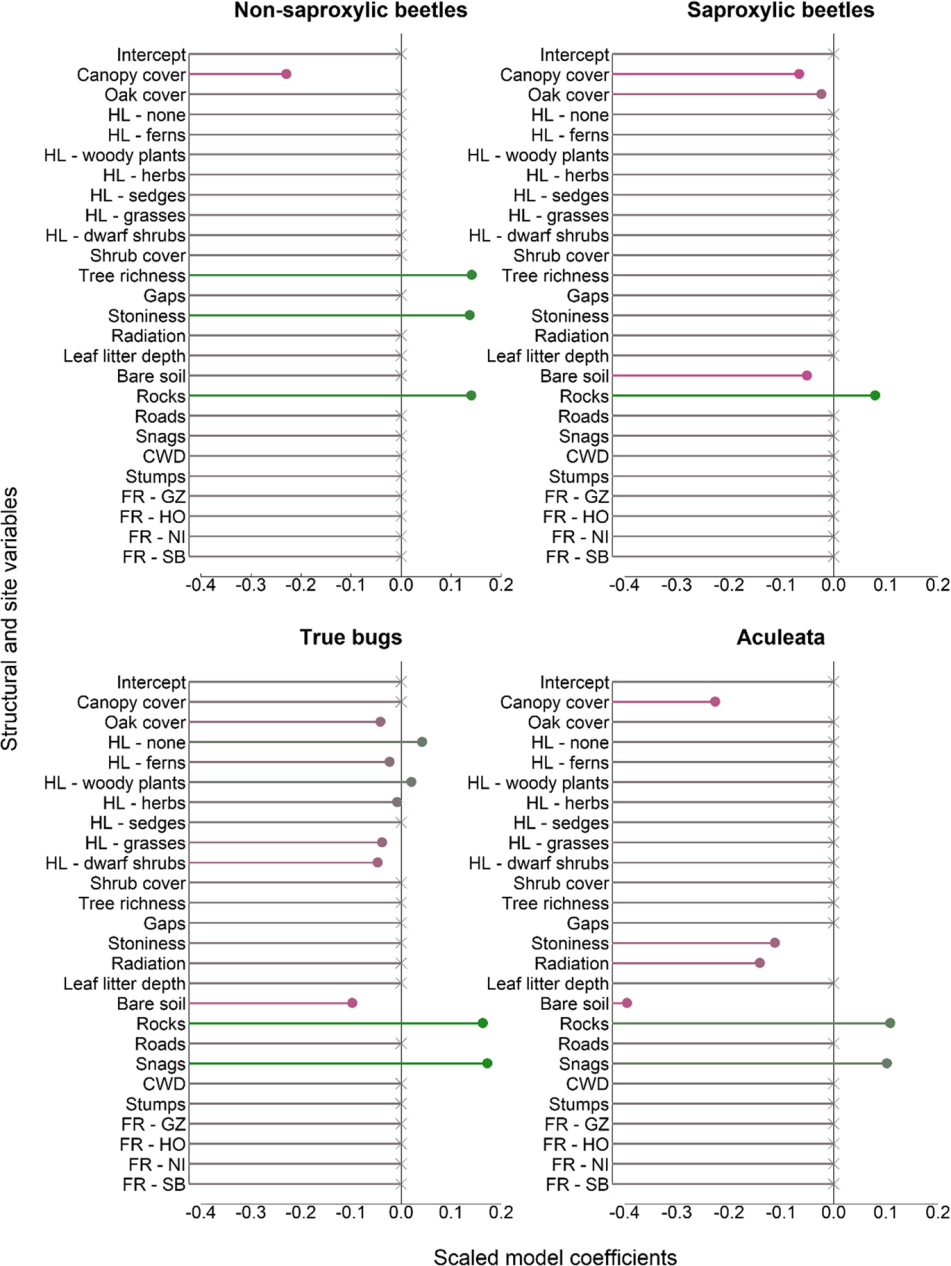
Values for the scaled model coefficients of the gradient boosted regression models are shown as a measure of effect size for species groups sampled in the understory layer (trunk eclectors/flight interceptions/window traps). A tenfold cross-validation was used to calculate the best model. Red (negative) or green (positive) line/point color illustrates the nature of relationships and their intensity between explanatory variables and arthropod group richness. No or very weak relationships (β < 0.001) are indicated by crosses at the end of the lines

The effect of the studied variables on species composition and richness was compared for the eight species groups subjected to an RDA analysis (ground floor: beetles, rove beetles, ground beetles, and spiders; understory tier: saproxylic beetles, non-saproxylic beetles, true bugs, and Aculeata). The number and identity of variables for which an effect could be detected differed between species richness (*n* = 12) and species composition (*n* = 10). For some variables, namely, cover % of bare soil, rocks, and amount of snags, an effect on species richness was found in some groups but none on species composition. Whereas shrub cover had an effect on species composition (true bugs), no effect was found for species richness. Variable importance differed greatly between species richness and composition for all groups. A noticeable exception was canopy cover (5 out of 8 groups), being a potentially important driver of both species richness and composition (Figs. 4 and 5).

### Is species richness correlated between arthropod groups?

**Fig. 6:**
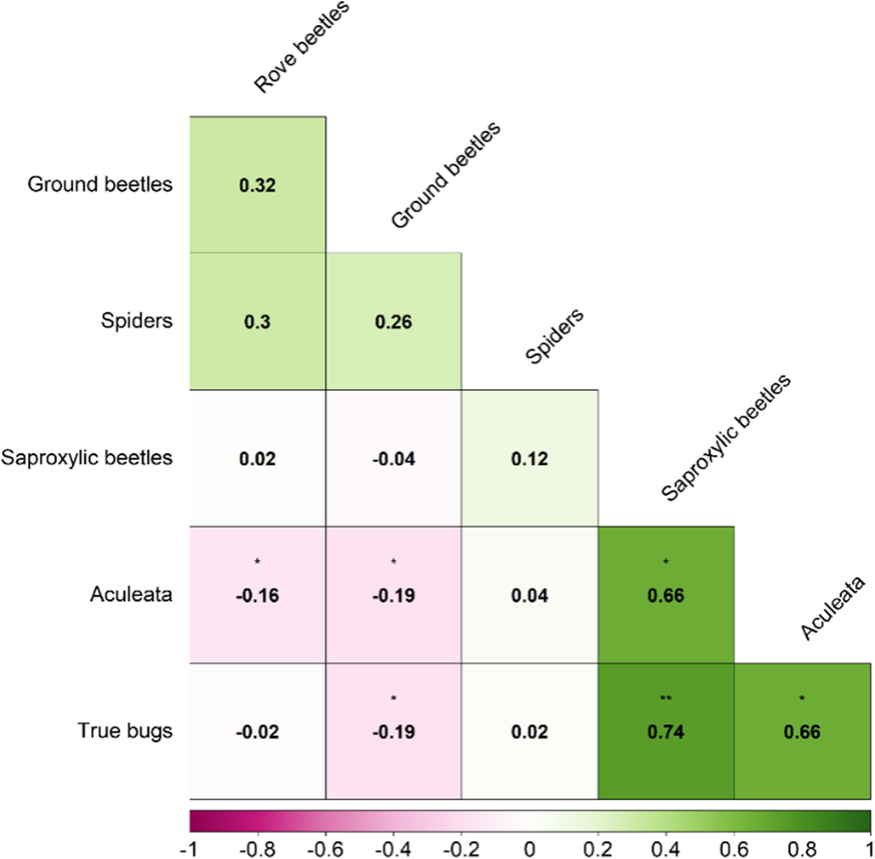
Spearman rank partial correlation matrix on species richness values for some species groups. The effect of the Strict Forest Reserve was partialled out. Negative correlations are illustrated in red and positive correlations in green. Correlation strength is indicated by color saturation. Associated* p* values are depicted as stars: 0.05 ≤ *, 0.01 ≤ **, 0.001 ≤ ***0.001. Ground beetles, spiders, and rove beetles were sampled with pitfall traps, saproxylic beetles, Aculeata and true bugs with eclector traps, and window traps or panes

To account for the strong influence of FR identity for some species groups, a partial correlation was applied (Fig. 6). In some cases, pitfall traps and the other (understory) trap types were not set up in the same sampling location. Mean species richness of the nearest two understory trap types to pitfall traps was used to test correlation between species groups. All species groups sampled in the ground layer (pitfall traps) showed a very weak positive, *r*(50) ≤ 0.32,* p* ≥ 0.31, but not significant correlation in species richness. The understory layer showed a strong positive correlation for saproxylic beetles and true bugs, *r*(50) = 0.73, *p* ≤ 0.02. Aculeata were weak to moderate positively correlated with the other groups of the understory, *r*(50) ≤ 0.74, *p* ≤ 0.05. Only weak significant negative relationships, *r*(50) ≤  − 0.19, *p* ≤ 0.05, between some species groups of both vegetation layers were detected. Mean species richness values of the nearest two understory trap positions from pitfall traps were used to calculate the Spearman partial rank correlation coefficient. The distance between pitfall and understory traps varied in some cases; they were at least 8.2 m and up to 81.2 m apart (28.6 ± 2.2 m).

### Which arthropod groups may be suitable for a trend or targeted monitoring?

**Fig. 7:**
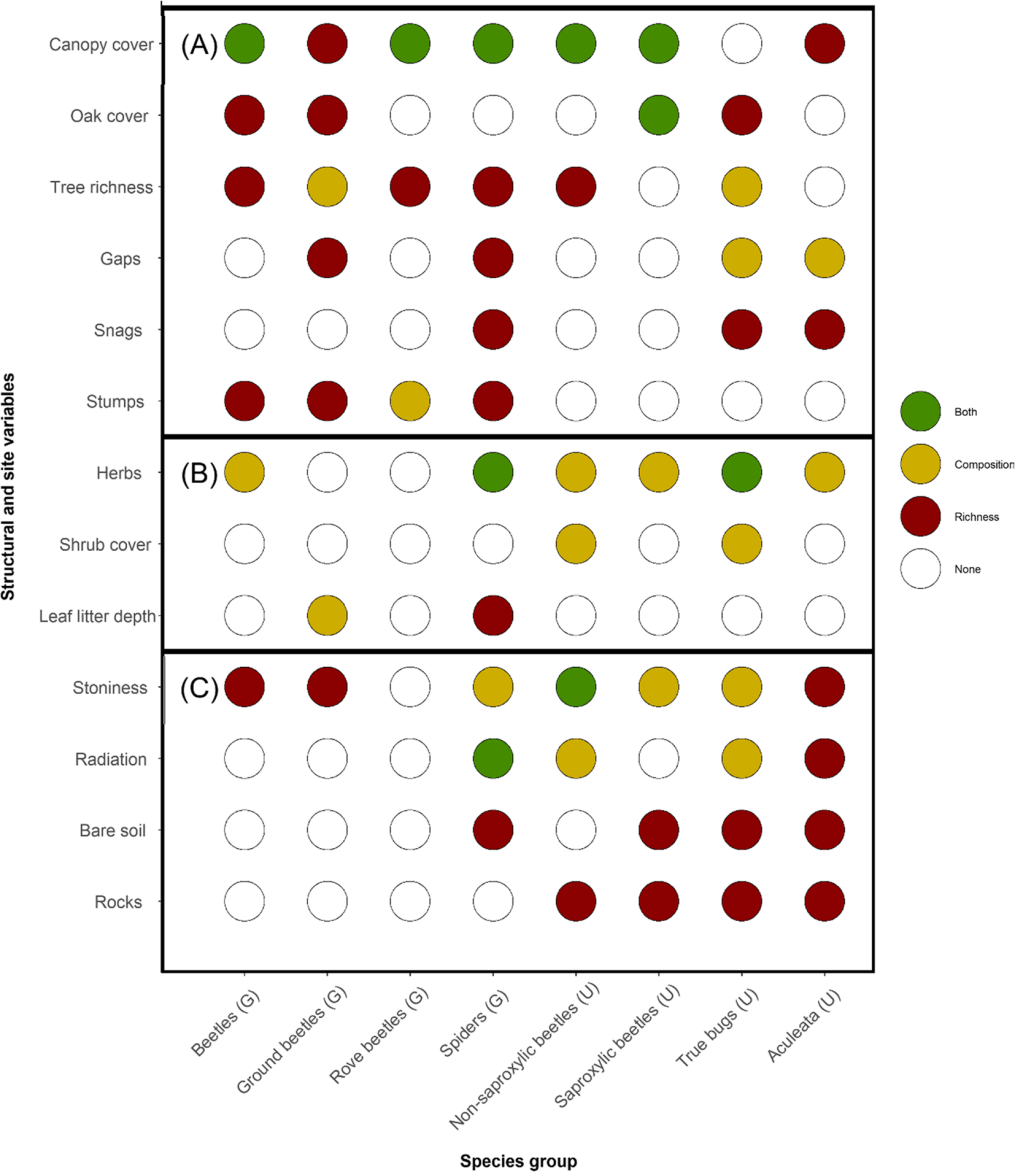
Structural, vegetation, and site variable importance are shown for five arthropod groups. Results of RDA and regression boosting models suggested that colored variables were important to either: species composition (yellow), richness (red), or both (green). Variables were grouped into three categories according to their likelihood of being influenced by management activities: (A) = direct effect, (B) = indirect effect, (C) = no effect

The potentially influential variables can not only be grouped into structural and site attributes but also according to their sensitivity to management as shown in Fig. 7. Some species groups such as spiders and true bugs seem to be driven by a wide range of variables (*n* = 10), affecting composition and richness differently. This finding is in contrast to rove beetles, which seem to respond only to a few of our tested variables, namely, canopy cover, tree richness, and quantity of stumps. Ground beetles reacted to five out of six analyzed variables directly related to management and only to one variable (stoniness) that depicts site conditions.

## Discussion

Beyond the specific findings in the data set, our approach shows a pathway to utilizing existing biodiversity survey data following adaptations to long-standing protocols. This is important because a 1:1 continuation of historical data sets into the future is not feasible and based on the research setting at the time of conceptualization, as compared with the present day, is also rarely sensible. The problems our ecosystems face today vary from those known and relevant decades ago. Yet these historical data are irretrievable and invaluable. We need to develop approaches to make use of them rather than ignoring these data sets and starting over.

### Which variables drive arthropod communities?

A large part of variation in the data set was left unexplained, which is very common in ecological studies (Zuur et al., 2007). General causes might be important unmeasured environmental variables or stochasticity in biological processes (Økland, 1999). In this study, the long time between the recordings of arthropods and site attributes most likely caused additional unexplained variation. Variable importance varied greatly between species groups.

Measures related to light availability and temperature, such as canopy cover or potential sun radiation, were important to all arthropod groups affecting either richness, composition, or both. Light availability and temperature determine amongst other things understory growth and diversity (Dormann et al., 2020; Gray et al., 2002). They have been repeatedly shown to either directly or indirectly affect arthropod communities (Černecká et al., 2020; Gossner, 2009; Seibold et al., 2016). Canopy cover, as a management-related variable, is often used as a proxy for light availability or temperature (Dormann et al., 2020). It is therefore interesting that the environmental variable potential solar radiation was also identified as an important driver of arthropod communities, pointing to differences in resource availability that cannot be influenced by management actions. Therefore, we suggest that both measures should be assessed either directly or indirectly for a causal large-scale monitoring program to establish initial site differences in solar radiation.

Our finding that some arthropod groups reacted to the presence of larger canopy gaps is in line with other studies for spiders (Perry et al., 2018), ground beetles (Heliölä et al., 2001), Aculeata (Braun-Reichert et al., 2021), and true bugs (F. Müller et al., 2008a, 2008b). Contrary to other studies (e.g., Lachat et al., 2016), we did not detect a relationship of canopy gaps with saproxylic beetles. A possible explanation was suggested by Sebek et al. (2015) who found that the positive correlation of saproxylic beetle diversity with larger canopy openings observed in their study might be caused by post-logging residues and stumps, whereas results of the permanent forest structural plots showed that deadwood was scarce in all our study sites (Schneider et al., 2021a, 2021b).

For some tested variables, namely, cover of roads (30 m radius) and coarse woody debris diversity (CWD), no effect on both species richness or composition could be detected. Results of a meta-analysis by Lassauce et al. (2011) suggest that there is a positive correlation between volume of deadwood and saproxylic beetle richness but that it might be only one of the key factors. Other studies found that deadwood placement on different spatial scales and quality can be more important than mere local deadwood volume (Økland et al., 1996; Vodka et al., 2009). Müller et al. (2015) for example observed in a multiscale study that higher temperatures are able to compensate for low amounts of deadwood. We found that one of the key factors for the saproxylic beetle composition was the dominant plant group of the herbaceous layer. This result might point to either to underlying differences in microclimatic conditions (moisture, sun exposure, and ground temperature) or in the availability of feeding plants for adults (Bouget et al., 2014).

The fact that we did not detect an effect of CWD on saproxylic species richness or composition in our study might also be caused by the general low amount of deadwood at the study sites. Another factor could be the limited accuracy of the retrospective approach. However, large deadwood objects present at the time of the faunistic sampling would have been detected because they require several decades to decay (Müller-Using & Bartsch, 2009; Rock et al., 2008). For smaller compartments such as branches, it can be assumed that the past amount does not differ much from the time of habitat assessment. We also investigated CWD diversity rather than volume, using a rather rough index. Therefore, we cannot conclusively determine what caused the missing relevance of snags and CWD for the saproxylic beetles in our study. It might be either one or a mix of the overall small amount and variability of deadwood or other factors being more important such as the dominant plant group (or correlated environmental variables, or the retrospective nature of this study).

The number of stumps is a more reliable measure in our study, as management ceased at least in the unmanaged site with the establishment of the FR. It can be treated as a simple measure of past management intensity (Kahl & Bauhus, 2014). Spider and ground beetle richness showed a weak negative effect, and the rove beetle community reacted strongly towards an increase in the number of stumps. This suggests that these groups might be sensitive to changes in management intensity, which is congruent to other studies or findings of meta-analyses (Junker et al., 2000; Paillet et al., 2010). Interestingly, the number of stumps had the strongest effect of all structural variables on ground-dwelling beetles associated with forests. Forest affinity patterns (i.e., forest specialists) of arthropod species can therefore be assumed to indicate the disturbance level of forests similar to plants (Schmidt et al., 2012). An effect of forest management on forest specialists has been observed by other studies. Schall et al. (2018) found that four out of eight studied species groups were affected by the spatial grains in which management was applied. Lange et al. (2014) reported differences in abundance ratios of forest specialists-to-open habitats for ground and rove beetles in managed and unmanaged forests. Structural attributes driving these results varied between these two species groups, but similar to our result, the overall largest driver was the location. Further research should be done to unravel this interesting relationship in more detail.

Another surprising result was the negative effect of oak trees on species richness. Oak trees were very rare in the study areas (only recorded in 3 sampling plots) which points towards a statistical artifact. The results of this study contradict strongly with existing knowledge (Brändle & Brandl, 2001; Mölder et al., 2019; Vogel et al., 2021) and might be a result of under-sampling these occurrences. This further underlines that sampling of rare habitat structures might require a different sampling protocol in addition to the ongoing monitoring.

### Are species richness and community composition driven by the same variables?

In our study, species richness and composition were driven by different variables. This is not surprising and has been found in other studies (Aggemyr et al., 2018).

Species composition provides a more complete picture of species assemblages than overall species richness. Species identity and their configuration pattern being an important part of species composition, even if using presence/absence data only (Aggemyr et al., 2018). The presence of species can also often directly be linked to environmental conditions, a fact which has been intensively used by the “character” species approach (Braun-Blanquet, 1932). Furthermore, two assemblages that hold the same number of species (species richness) could theoretically be home to a completely different set of species. Accordingly, species richness and composition differ in the information they provide about species assemblages. Important variables for species richness models might especially affect rare species, while variables identified for species composition in our study might be driven by specific species or species groups being connected to site variables independent of species dominance (presence-absence data). Ideally, both aspects of arthropod communities should be investigated to interpret monitoring results meaningfully.

### Is species richness correlated between arthropod groups?

As in other studies, generally low correlation for species richness between different arthropod groups was observed (Westgate et al., 2014). Notable exception was the strong correlation between true bugs, saproxylic beetles, and Aculeata. A possible explanation could be that a range of variables affected all three of these species groups (e.g., rocks, cover % of bare soil, and canopy cover).

### Which arthropod groups may be suitable for a trend or targeted monitoring?

Different subgroups of ground-dwelling beetles reacted differently to explanatory variables. This suggests that examining meaningful subgroups (e.g., along functional traits such as feeding or habitat requirements) during analysis, rather than “the beetles,” would enhance the ability to detect significant relationships in monitoring data.

Certain variables sampled in this study, i.e., canopy cover, tree species richness, gaps, stump quantity, or deadwood diversity are strongly affected by management actions. Others, such as the dominant plant group in the ground layer, can be indirectly affected by management but are also site-dependent.

Species groups chosen for targeted monitoring should be sensitive to specific ecosystem processes (Sparrow et al., 2020), e.g., structural changes by forest management. Our results showed that some species groups such as true bugs and spiders are sensitive to a broad range of site or forest structural variables. From the sensitivity perspective, this makes them good groups for general trend monitoring (e.g., effects of climate change). Spiders have already been suggested for biodiversity trend monitoring in German forests (BfN. (2021)). However, both groups might be at this taxonomic resolution suboptimal for research questions focusing directly on the effect of forest management on biodiversity due to their broad indicator range. A broad indicator range has its perks such as easy integration of new research questions but also an important drawback if focusing on the whole community during analysis. To entangle the underlying causes of changes in richness or composition in these species groups, a large set of environmental and structural drivers together with an extensive sample size of traps and individuals have to be assessed to ensure statistical power. This issue of masking important underlying trends when using overall species community data has been noted before (e.g., Staab et al., 2023). Therefore, we suggest focusing on functional or taxonomic subgroups during the analysis of these species groups. To identify the subgroups that are most useful here requires further investigation.

Our results imply that rove beetles and ground beetles could be a good indicator choice for forest management-related questions, as they mainly reacted to variables that can be influenced by management. Contrary, it should not be concluded from our findings that saproxylic beetles can be discarded from management-focused monitoring programs. The limited relationships found with management-related variables (in particular deadwood) in our study are most likely caused either by our study design or specific site conditions. Other studies observed strong indications of these relationships, especially with different deadwood measures (e.g., Bouget et al., 2013).

Last but not least, only a limited number of variables were tested in this study. Sensitivity to targeted processes is just one part of a range of requirements such as ease of identification, cost-effectiveness, and complementarity in their indicative power, suggested by a vast amount of literature (Heink & Kowarik, 2010; Noss, 1990, 1999; Pereira et al., 2013) for an indicator group to be suitable.

### The structural sampling protocol

Some adjustments to the initial sampling protocol were made for its future application, based on experiences made during data analysis. As already mentioned, very rare occurrences of variables such as rare habitats, or rare tree species cannot be meaningfully analyzed with this sampling method. Larger plot radii for bordering rare or land use habitats should be recorded. Presence–absence sampling will be substituted with real estimations/measurements; this is obvious for deadwood but also distances from rare habitats/bordering land uses should be recorded. Also, the hypotheses that the stand development phase indicates important stand structural measurements such as diameter, height distributions or maximum diameter, and number of microhabitats should be explored in more detail. In this study, the forest stands in the FR were very similar to their development phases. Therefore, the effect of the forest development phase could not be analyzed meaningfully; direct measurements, however, might have been able to show differences in arthropod diversity or composition in forest stands.

## Conclusions

We demonstrated that it is possible to utilize existing biodiversity survey data and adjust long-standing protocols. Although, in our case, there are strong restrictions due to the long time between species and structural attribute sampling. A re-assessment of these plots and trap sites will build a time series of arthropod richness and community composition over several decades, making it a unique study. Our results show that the sole focus on species richness as a measure of ecosystem condition in biodiversity monitoring is not sufficient. Species richness only provides a simplified picture of species assemblages, and important changes might be only apparent in compositional patterns. We also suggested that trait (e.g., saproxylic) or habitat use (e.g., forest affinity) grouping of species will allow for rather concrete deductions in respect to the role of forests and their management in biodiversity conservation, a finding that can aid the further development of conservation programs. In conclusion, we would like to encourage committing more effort in adaptive monitoring processes and with that being able to fully utilize data from long-standing monitoring programs. Monitoring questions will continue to change over time, and it is just not efficient to start with a clean slate every time this happens. It is a fine line between developing long-time programs in a way that allows future shifts in focus, add-ons, or changes in methodology and at the same time to not overcommit in sampling intensity and extend “just in case.”

### Supplementary Information

Below is the link to the electronic supplementary material.

Supplementary file1 (DOCX 372 KB)

Supplementary file2 (DOCX 1.58 MB)

## Data Availability

The datasets generated for this study are available from the SGN (Steffen Pauls) and the NW-FVA (Peter Meyer) upon reasonable request.
