# Supplementary material for: Adaptive monitoring in action—what drives arthropod diversity and composition in central European beech forests?
Source: Environ Monit Assess. 2024 Apr 24;196(5):470. doi: 10.1007/s10661-024-12592-4 (PMC11043153; doi:10.1007/s10661-024-12592-4)
Supplement: Supplementary file 1 — Supplementary file1 (DOCX 372 KB) [file 10661_2024_12592_MOESM1_ESM.docx]

**Supporting Information 1**

**Article title:** Adaptive monitoring in action – what drives arthropod diversity and composition in central European beech forests

**Journal name:** Environmental Monitoring and Assessment

**Authors:** Constanze Keye*^1^, Marcus Schmidt^1^, Christian Roschak^1,6^, Wolfgang H. O. Dorow^2^, Viktor Hartung^2,3^, Steffen U. Pauls^2,4^, Alexander Schneider^2,4^, Christian Ammer^5^, Laura Zeller^5^, Peter Meyer^1^

**Affiliations:**

^1^ Department for Forest Nature Conservation, Northwest German Forest Research Institute, Prof.-Oelkers-Str. 6, 34346, Hann. Münden, Germany

^2^ Senckenberg Research Institute and Natural History Museum Frankfurt, Senckenberganlage 25, 60325, Frankfurt am Main, Germany

^3^ LWL-Museum of Natural History - Westphalian State Museum with Planetarium, Sentruper Str. 285, 48161, Münster, Germany

^4^ Institute of Insect Biotechnology, Justus-Liebig-University, Heinrich-Buff-Ring 26-32, 35392, Gießen, Germany

^5^ Department of Silviculture and Forest Ecology of the Temperate Zones, University of Göttingen, Büsgenweg 1, 37077, Göttingen, Germany

^6^ New Zealand Forest Research Institute Ltd (Scion), Te Papa Tipu Innovation Park Tītokoran gi Drive, 3020, Rotorua, New Zealand

* **Corresponding author**

CK, constanze_keye@icloud.com

Table S1

Number of sampling plots are shown for the individual research areas. The ground tier was sampled using pitfall traps. Trunk eclectors, flight interception traps and window traps were employed to sample the understory tier.

| Study area |  | Ground tier | |  | Understory tier | |
| --- | --- | --- | --- | --- | --- | --- |
|  |  | SFR | MRA |  | SFR | MRA |
| Goldbachs- und Ziebachsrück |  | 8 | 7 |  | 9 | 5 |
| Hohestein |  | 6 | 6 |  | 10 | 5 |
| Niddahänge östlich Rudingshain |  | 8 | 6 |  | 14 | 10 |
| Schönbuche |  | 6 | 6 |  | 12 | 4 |

Table S2

Averaged distances (m) between trap sites are shown for all research areas. The ground tier was sampled using pitfall traps. Trunk eclectors, flight interception traps and window traps were employed to sample the understory tier, in some cases they were independently places from the pitfall trap sites.

|  | mean | min | max |
| --- | --- | --- | --- |
| Ground tier | 135 | 41 | 260 |
| Understory tier | 165 | 42 | 362 |


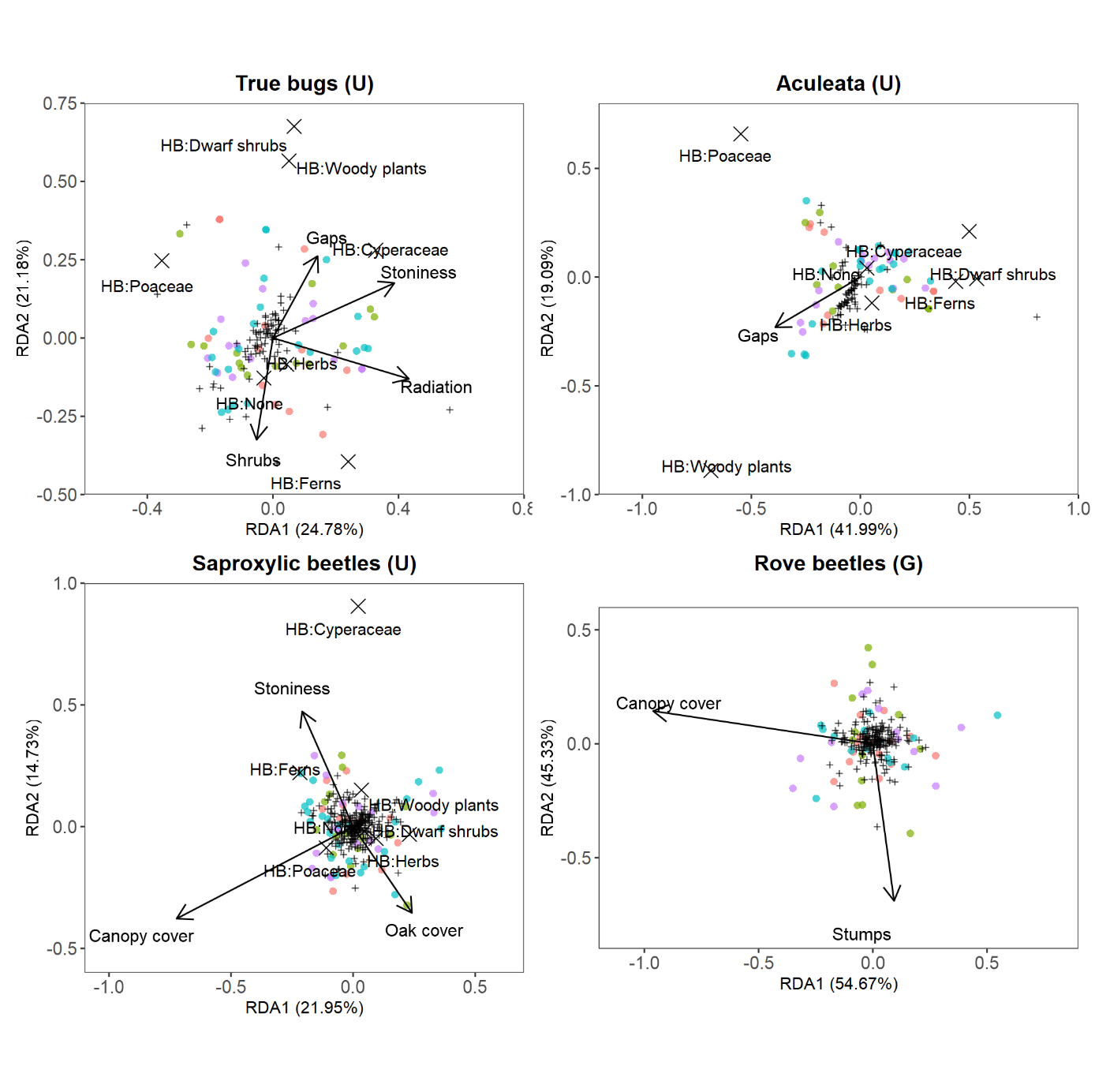


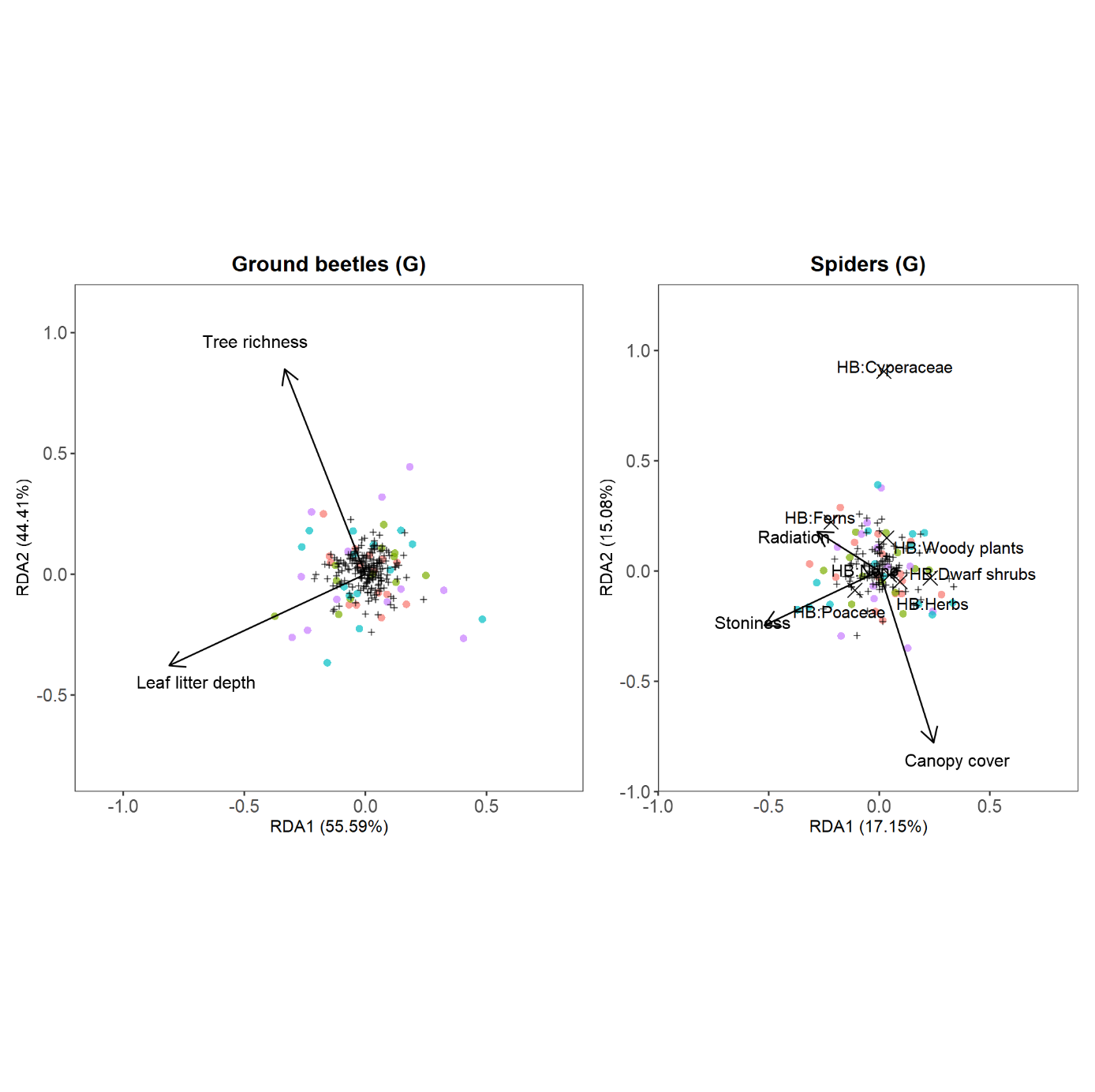


Figure S3 Partial tb-RDA triplots (correlation plot/scaling 2, WA scores) for selected taxonomic or functional arthropod groups of the ground floor (G) and the understory layer (U). Only the environmental variables which have been selected by the stepwise forward selection process are displayed. Proportions of the total explained variance by the first two canonical axes are shown (RDA1, RDA2). Sites are symbolized by *points* based on the weighted averages of species scores (WA scores) which are more robust to noise in comparison to other methods (McCune, 1997). *Point color* reflects the affiliation to different strict nature reserves: *orange*=Goldbachs- und Ziebachsrück, *blue*= Niddahänge östlich Rudingshain, *green*=Hohestein, *pink*=Schönbuche. The plus signs depict arthropod species, crosses show centroids of factor variables. Length and direction of arrows indicate the direction and rate of steepest increase of the variables.
