# Supplementary material for: Adaptive monitoring in action—what drives arthropod diversity and composition in central European beech forests?
Source: Environ Monit Assess. 2024 Apr 24;196(5):470. doi: 10.1007/s10661-024-12592-4 (PMC11043153; doi:10.1007/s10661-024-12592-4)
Supplement: Supplementary file 2 — Supplementary file2 (DOCX 1.58 MB) [file 10661_2024_12592_MOESM2_ESM.docx]

**Supporting Information 2**

**Article title:** Adaptive monitoring in action – what drives arthropod diversity and composition in central European beech forests

**Journal name:** Environmental Monitoring and Assessment

**Authors:** Constanze Keye*^1^, Marcus Schmidt^1^, Christian Roschak^1,6^, Wolfgang H. O. Dorow^2^, Viktor Hartung^3^, Steffen U. Pauls^2,4^, Alexander Schneider^2,4^, Christian Ammer^5^, Laura Zeller^5^, Peter Meyer^1^

**Affiliations:**

^1^ Department for Forest Nature Conservation, Northwest German Forest Research Institute, Prof.-Oelkers-Str. 6, 34346, Hann. Münden, Germany

^2^ Senckenberg Research Institute and Natural History Museum Frankfurt, Senckenberganlage 25, 60325, Frankfurt am Main, Germany

^3^ LWL-Museum of Natural History - Westphalian State Museum with Planetarium, Sentruper Str. 285, 48161, Münster, Germany

^4^ Institute of Insect Biotechnology, Justus-Liebig-University, Heinrich-Buff-Ring 26-32, 35392, Gießen, Germany

^5^ Department of Silviculture and Forest Ecology of the Temperate Zones, University of Göttingen, Büsgenweg 1, 37077, Göttingen, Germany

^6^ New Zealand Forest Research Institute Ltd (Scion), Te Papa Tipu Innovation Park Tītokoran gi Drive, 3020, Rotorua, New Zealand

* **Corresponding author**

CK, constanze_keye@icloud.com


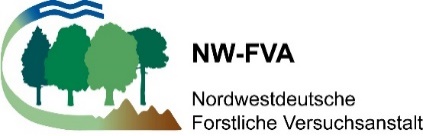


FIELD MANUAL

**Vegetation structure and habitat mapping**

**of Hessian natural forest reserves**

**for monitoring of insect populations**

Stand: November 2021


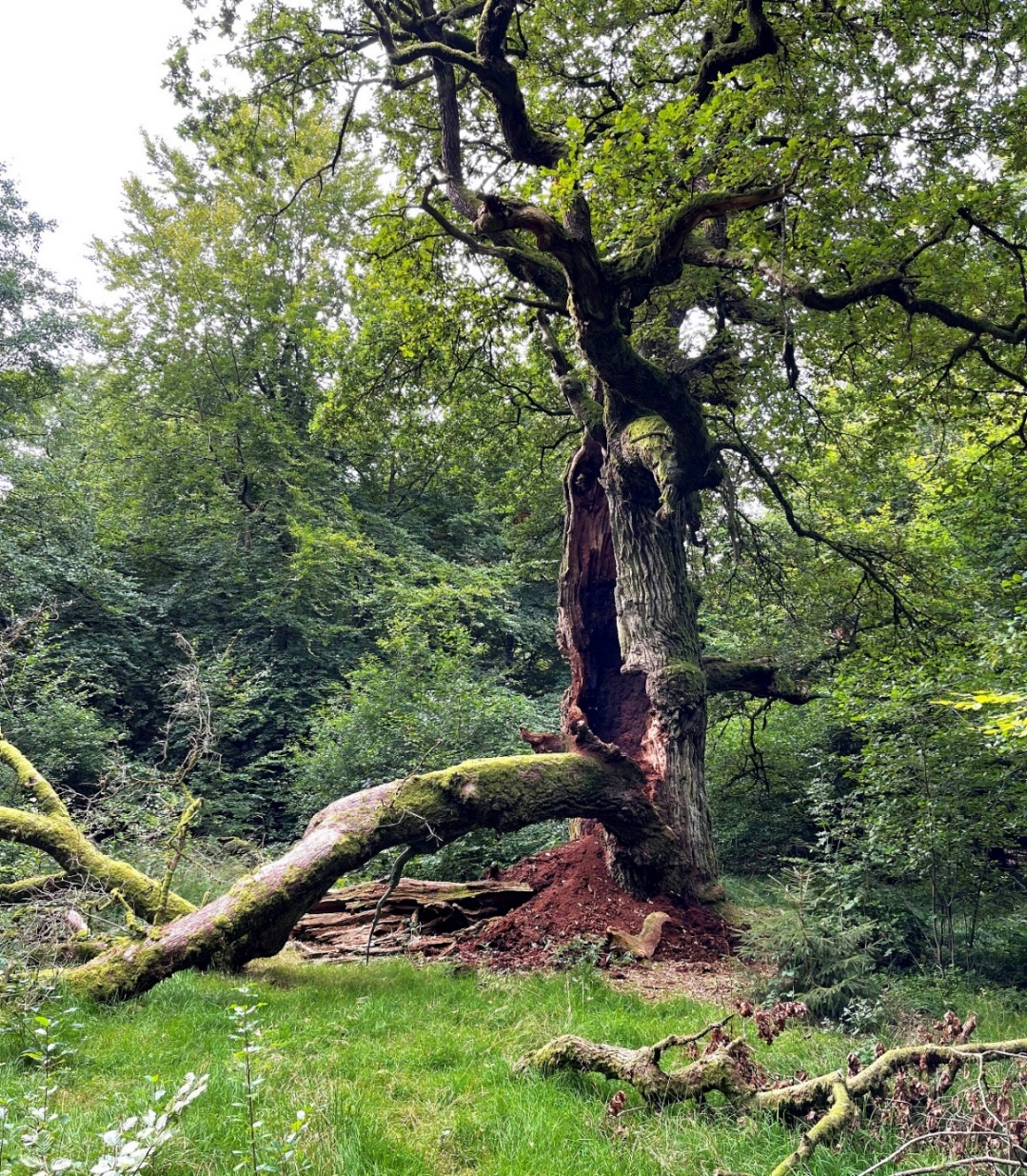


Peter Meyer, Marcus Schmidt, Katja Lorenz, Christian Roschak, Constanze

Keye

Nordwestdeutsche Forstliche Versuchsanstalt
 Abteilung Waldnaturschutz

Professor-Oelkers-Straße6

34346 Hann. Münden

www.nw-fva.de

| Abbreviations |  |
| --- | --- |
| BHD | Breast height diameter (1.3 m) |
| D1 / D2 | Diameter 1 / Diameter 2 |
| DC | Datacollector in FieldMap |
| FM | FieldMap |
| GIS | Geographical Information System |
| GPS | Global positioning system |
| ID | Identification number |
| MPKT_D1 | Measuring point diameter 1 |
| MPKT_D2 | Measuring point diameter 2 |
| Navi-Plot | Navigation plot |
| NW | Natural forest |
| NWR | Natural forest reserve |
| OZ | Order number |
| PM | Project Manager FieldMap Software |
| PKR | Sampling plot |
| TR | Strict forest reserve |
| VF | Unmanaged comparison site |

Cover picture: Veteran oak tree on ancient wood pastures in the primeval forest Sababurg near Hofgeismar, district Kassel, Hesse (C. Roschak)

Table of Contents

[Figures 4](#_Toc139440496)

[Tables 4](#_Toc139440497)

[1. Introduction 5](#_Toc139440498)

[2. Sampling Procedure 5](#_Toc139440499)

[2.1. Locating the trap sites 5](#_Toc139440500)

[2.2. Marking the trap locations 6](#_Toc139440501)

[2.3. Trap types and sampling 6](#_Toc139440502)

[2.4. Field equipment 6](#_Toc139440503)

[3. Trap site attributes 7](#_Toc139440504)

[3.1. General trap site information 7](#_Toc139440505)

[3.2. Stand structure 11](#_Toc139440506)

[3.3. Forest development stages 12](#_Toc139440507)

[3.4. Species composition of tree and shrub and herbaceous layer 12](#_Toc139440508)

[3.5. Deadwood 14](#_Toc139440509)

[3.6. Linear or small-scale habitats and structures 16](#_Toc139440510)

[3.7. Eclector trees 17](#_Toc139440511)

[4. Literature 20](#_Toc139440512)

[5. Appendices 21](#_Toc139440513)

[Appendix 1 - Species list 21](#_Toc139440514)

[Appendix 2 –Habitat types 25](#_Toc139440515)

# Figures

[Figure 1. Field data sheet with information on the trap locations 6](#_Toc139279074)

[Figure 2. Soil texture triangle showing the German soil textures classes 9](#_Toc139279075)

# Tables

[Table 1. Classification of the terrain form 8](#_Toc139279140)

[Table 2. Abbreviations for the different humus forms 8](#_Toc139279141)

[Table 3. Classification of the volume proportion of stones in the topsoil 9](#_Toc139279142)

[Table 4. Percentage cover classes used for assessing different forest attributes. 10](#_Toc139279143)

[Table 5. Occurrence classes of an attribute 10](#_Toc139279144)

[Table 6. Different species groups of the herb layer 11](#_Toc139279145)

[Table 7. Classification of forest development stages at the trap site. 12](#_Toc139279146)

[Table 8. Decomposition classes of deadwood 14](#_Toc139279147)

[Table 9. Chart for determining the condition type of the eclectic trees 17](#_Toc139279148)

[Table 10. Description of microhabitat classes 19](#_Toc139279149)

# Introduction

Natural forest research extends the knowledge about natural processes in forest landscapes. The outcomes gained are especially important to develop well designed close to nature forest management concepts. Closely linked to forest stand structures are a wide variety of species groups. Forest management creates disturbances that have a wide impact on forest structures at different scales. The effects on species composition and diversity especially that of insects can be therefore immense, and at a very small scale. For this reason, in addition to broader scale forest structure and habitat mapping at stand level (chapter 2), information on a smaller - trap level scale (chapter 3) is collected here.

Over all Hessian strict forest reserves and their comparison sites a sampling grid, with permanent fixed-area sampling plots were established. At these plots forest structural data is recorded for natural forest research. For all sampling plots, coordinates are available in UTM (plus shape file). Plot centres are also marked in the field with a white or yellow steel cap attached at ground level (in rare cases, this cap may have been removed by wild boars). The sampling plot number can be found on top of the centre cap.

# Sampling Procedure

## Locating the trap sites

The angle (degree) and distance (m) of the individual trap locations are measured from a selected reference structural fixed-area sampling plot. If 2 or more trap types are set up at one location, only one measurement of angle and distance is required.

In the case of pitfall traps, mostly 3 traps were placed in a row. In rare cases there are only two or one (Fig. 1). The pitfall traps always have a distance of 5 meters between each other and the angle of the trap row starting from the first trap is also specified in the field data sheet. If there is a row of 3 traps, the middle trap is taken as the reference point for the forest structural mapping. If there are only 2 traps, the centre between them is taken as the reference point (2.5m in angular direction). If mixed trap types are present, the middle pitfall trap establishes the plot centre, if no pitfall trap is present – plot centre can be chosen close to a trap type of choice, but has to be recorded.


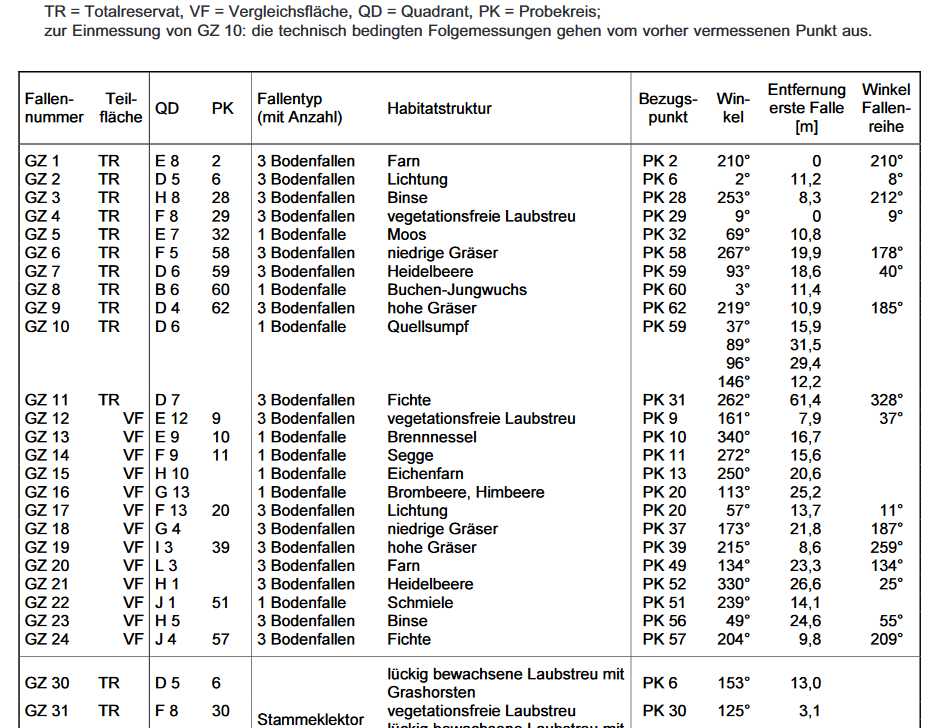


Figure 1. Field data sheet with information on the trap locations

## Marking the trap locations

Sampling has to be preferably carried out in spring or summer. If this is not possible, an additional field session has to be undertaken during these seasons, as it is not possible to record all attributes during winter. The trap locations should therefore be temporarily marked with flagging tape attached to the nearest tree and a tonking stick (bamboo stick) at the plot centre.

## Trap types and sampling

Different arthropod trap types are deployed, each with different installation/mounting methods. For all trap types a basic set of the forest structural and habitat attributes (see section 3 ; OZ 1 -63 ) are recorded (once for each trap site, regardless of how many different traps were installed). For the eclector trap types (e.g. trunk, or stump eclector), additional information of the trap tree) is required (see section 3.7, OZ 64 - 66).

## Field equipment

- Getac tablet with Fieldmap project
- Garmin GPS device
- Compass (degree)
- Haglof Postex Rangefinder with monopod staff
- 50m tape measure
- Ranging pole
- 80cm caliper
- Flagging tape
- Spade

# Trap site attributes

## General trap site information

The recording of the general plot characteristics is carried out at the beginning of the inventory. The attributes should not simply be updated during subsequent inventories, but must be checked again. In principle, all attributes are surveyed on an area within a 10 m radius around the plot centre, unless different plot radii are defined for the individual attributes.

OZ 1: Strict forest reserve (*NWID*): identification number and name

OZ 2: Area type (*Information*): strict forest reserve or comparison site

OZ 3: Geographical latitude (*RW*): The information has been already collected and is available as easting in the UTM coordinate system. However, if traps cannot be located at the available coordinates, these have to be amended. Correct coordinates can be calculated using angle and distance to the next forest structural sampling plot (already recorded). Recording in the field is not necessary.

OZ 4: Geographical longitude (*LW*): Data is available as northing in the UTM coordinate system. A conversion to WGS84 is possible in GIS if required. In case that the pre-recorded coordinates are incorrect, the same procedure as explained in OZ 2 has to be applied.

OZ 5: Trap ID (*Fallen_Nr*): identification number of the individual trap or the trap triplet for pitfall traps. These trap numbers are automatically the trap site ID. If several trap types are present at a trap site, each trap type has to be recorded with a unique ID. Trap site information have to be noted only once, but replicated for each unique trap ID present at a specific trap site.

OZ 6: Trap type (Fallentyp): the type of trap set up at this specific site.

OZ 7: Inclination (*Neig_Polar*): the angle of slope between the reference forest structural sampling plot (grid point) and the trap site is measured, to enable a horizontal projection of the trap sites in the QGIS shapefile later. If the trap sites are located via an auxiliary point, 2 angles must be measured. First from the forest structural sampling plot to the auxiliary point and then from the auxiliary point to the trap site. The slope is measured in percent.

OZ 8: Topography (*GFORM*): the dominant terrain form in a 10m radius from the trap site centre (Table 1).

Table 1. Classification of the terrain form

| ID_TEXT | Value |
| --- | --- |
| PLAT | Plateau |
| KUPP | Back, crest, saddle, rib |
| OHAN | Upper slope |
| MHAN | Slope, middle slope |
| UHAN | Underhang |
| BOES | Embankment |
| TAL | Valley, stream bottom, hollow, gully, depression |
| TSCH | Valley closure |
| EBEN | Level |

OZ 9: Slope (*hneig*): The slope of the terrain measured in degree. The measurement is taken from the trap site centre using an inclinometer.

OZ 10: Aspect (*expo*): The aspect is estimated in degrees deviating from north using a compass.

OZ 11: Height above sea level (*hnn*): The information is derived from an elevation model in GIS and is not recorded in the field.

OZ 12: Humus form (*humusf*): The main humus form found within a 10m radius of the trap site is assessed (Table 3). For this purpose, 3 samples are taken and the most frequent humus form is recorded. The humus classification follows the guidelines of the Arbeitskreis Standortskartierung (1996, pp. 92-93). An English explanation of humus forms used can be found in Baritz (2003).

Table 2. Abbreviations for the different humus forms

| ID_TEXT | Value |
| --- | --- |
| LMULL | L-mull |
| FMULL | F-mull |
| MMOD | Mull-like moder |
| MOD | Moder |
| RHMOD | Moder-like Mor |
| ROHH | Mor |
| TORF | Peat |

OZ 13: Thickness of the humus layer (*humuscm*): The thickness of the humus layer in cm is estimated. Here, all 3 organic layers (O_L_= litter layer, O_F_= fermentation layer_,_ O_H_= humification layer) are combined and described in one value. According to the international nomenclature (Soil Science Society of America, 2001) these layers correspond to the following horizons: Oi (O_L_), Oe (O_F_), Oa (O_H_).

OZ 14: Soil type (*bodart*): The predominant soil type within a 10 m radius of the trap site and to a depth of 10 cm (starting beneath the humus layer) according to the specifications (Table 4, Fig. 2) given by the Arbeitskreis Standortskartierung (2016, pp. 77-80) and the [Bodenkundlichen Kartieranleitung](https://de.wikipedia.org/wiki/Bodenkundliche_Kartieranleitung) (Ad-Hoc-AG Boden 2005, p.142). Three soil samples are taken with the spade within the 10 m sampling plot radius and the most frequent soil form is recorded.


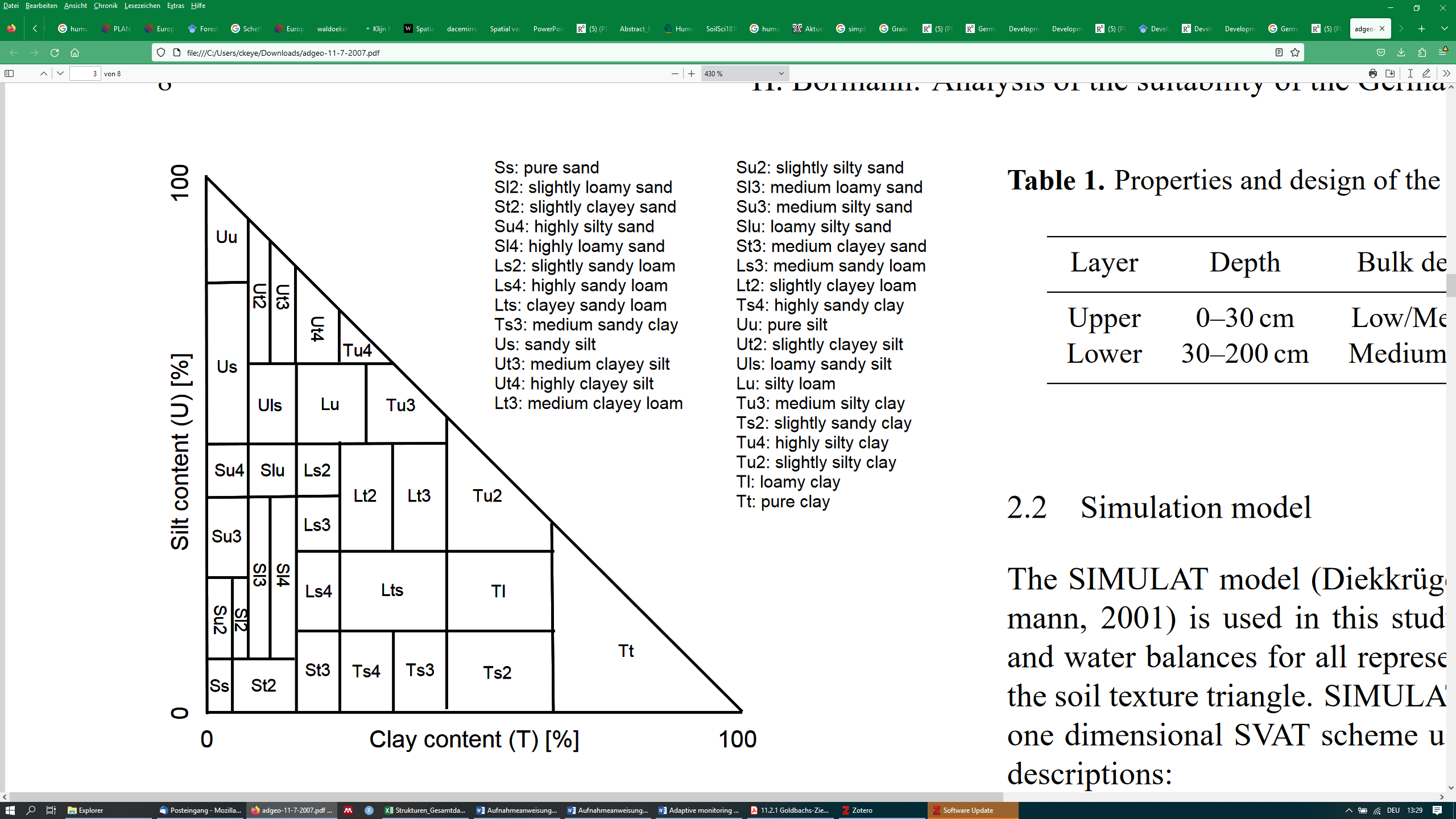


Figure 2. Soil texture triangle showing the German soil textures classes according to Ad-Hoc-AG Boden (2005) translated into English and redrawn by Bormann 2007.

OZ 15: Stoniness of topsoil (*skel*): The predominant skeletal content of the topsoil within a 10 m radius of the trap site and up to 10 cm soil depth (starting underneath the humus layer) according to the specifications by the Arbeitskreis Standortskartierung (2016, p. 355) (Table 3)

Table 3. Classification of the volume proportion of stones in the topsoil

| **ID_TEXT** | **Value** | **Volume (%)** |
| --- | --- | --- |
| 0 | No stones | 0 |
| 1 | very weakly stony, gravelly, grusig | < 2 |
| 2 | weakly stony, gravelly, grusig | 2 - 10 |
| 3 | medium stony, gravelly, grusig | 10 - 25 |
| 4 | strongly stony, gravelly, grusig | 25 - 50 |
| 5 | very strongly, stony, gravelly, grusig | 50 - 75 |
| 6 | Stones, gravel, grus | > 75 |

OZ 16: Leaf litter (*streutyp*): The predominant leaf litter type in a 10 m radius around the trap site: broadleaf, coniferous, or mixed litter.

OZ 17: Leaf litter cover (*streudeck*): Percentage of the 10 m sampling plot covered with leaf litter. The coverage ratio is estimated in the 10 m sampling plot according to Table 4.

Table 4. Percentage cover classes used for assessing area covered in relation to the total sampling plot area for different forest attributes.

| **ID_TEXT** | **Cover Percentages** |
| --- | --- |
| 0 | missing |
| 1 | < 5% |
| 5 | 5 - 25% |
| 25 | 26 - 50% |
| 50 | 51 - 75% |
| 75 | >75% |

OZ 18: Leaf litter depth (*streucm*): Thickness of the L-layer (largely undecomposed litter) estimated to full centimetres.

OZ 19: Bare soil (*mboden*): Cover percentage of open mineral soil patches without leaf litter cover within the 10 m sampling plot according to Table 4.

OZ 20: Sealed area (*versieg*): Cover percentages of sealed surfaces within a 10 m radius of the trap location according to Table 4.

OZ 21: Gravel and stones (*kies*): Total cover percentage for all rocks of a size from 2 to 200 mm. The cover is estimated in the 10 m sampling plot radius around the trap site centre according to Table 4.

OZ 22: Rocks (*block*): Total cover percentage for all rocks of a size of 200 and up to 630 mm. The classification is made in a 10 m radius around the trap location according to Table 5.

Table 5. Occurrence classes of an attribute

| **ID_TEXT** | **Value** |
| --- | --- |
| 0 | missing |
| 1 | single |
| 2 | frequent |

OZ 23: Boulders (*felsen*): Total cover percentage for all boulders from 630 mm and larger. The classification is made in a 10 m radius around the trap location according to Table 5.

## Stand structure

OZ 24: Cover of the 1st tree layer (*deckbs1*): The first tree layer is formed when the of a forest stand are clearly separated into 2 tree layers. This layer consists of the dominant and predominant trees of a stand (main layer and intermediate canopy layer participating in or adjacent to the upper canopy).

Observation area: 10 m radius around the trap site centre.

Cover percentage is estimated in classes according to Table 4.

OZ 25: Cover of the 2nd tree layer (*deckbs2*): In a two-layer forest the second tree layer is made up by understory trees (understory = below ½ top height, BHD ≥ 7cm).

Observation area: 10 m radius around the trap site centre.

Cover is estimated in classes, as for the tree layer 1 (Table 4).

OZ 26: Cover of the shrub layer (*deckss*): The shrub layer is formed by all woody plants (trees and shrubs) over 0.5 m in height and with a BHD < 7 cm.

Observation area: 10 m radius around the trap site centre.

Cover classes are estimated according to values given in Table 4.

OZ 27: The four most dominant species groups of the herb layer (*artgrks1 - artgrks4*): The herbaceous layer is composed of all herbaceous plants and woody plants up to 0.5 m in height.

Observation area: 10 m radius around the trap site centre

Selection of the predominant, second most abundant, third most abundant and fourth most abundant species group of the herbaceous layer according to Table 6.

Table 6. Different species groups of the herb layer

| ID_TEXT | Value |
| --- | --- |
| KRAUT | Herbaceous plants |
| FARNE | Ferns |
| SÜßGR | Poaceae |
| SAUER | Cyperaceae |
| ZWERG | Dwarf shrubs |
| GEHOE | Woody plants |

OZ 28: Cover percentage of the 4 most important species groups of the herb layer (*deckks1 - deckks4*): The classification of the cover of the 4 most important species groups occurring in the herb layer is analogous to the classification of the tree layer cover (Table 4).

Observation area: 10m radius around the trap site centre. Cover overlap of different sized plant groups may result in a total cover exceeding 100%.

OZ 29: Cover percentage of the moss layer (*deckms*): The moss layer is formed by all ground dwelling mosses and lichens.

Observation area: 10m radius around the trap site centre.

Cover percentage is estimated in classes given in Table 4.

## Forest development stages

OZ 30: Gaps in the enclosure ring between 10 and 30 m (*luecke30m*): Presence/absence of canopy gaps. Large canopy gap which have to be closed by regenerating trees rather than by neighbouring trees) in the enclosure ring around the trap site centre (radius 10 - 30 m). Recorded only as yes/no statement.

OZ 31: Forest development stage of tree layer 1 (*wepbs1*): See definition of 1st tree layer (OZ 24).

Observation area: 10 m radius around the trap site centre.

The classification into forest development stages is done according to the specifications in Table 7.

Table 7. Classification of forest development stages at the trap site according to the Hessische Anweisung für Forsteinrichtungsarbeiten (HMUELF 2002).

| ID_TEXT | Value |
| --- | --- |
| JW | Young growth: Regenerating trees until the canopy is closed |
| DI | Thicket: stand until the beginning of natural self-pruning. |
| ST | Pole timber: diameter at breast height (BHD) between 7 and 25 cm |
| GB | Small sawlog: BHD between 26 and 35 cm |
| MB | Medium sawlog: BHD between 36 and 50 cm |
| SB | Strong sawlog: BHD between 50 and 80 cm |
| SSB | Very strong sawlog: BHD > 80 cm |

OZ 32: Forest development stages 2nd tree layer (*wepbs2*):

See definition of 2nd tree layer (OZ 25).

Observation area: 10 m radius around the trap site centre.

Definition of possible forest development stages are given in Table 7.

## Species composition of tree and shrub and herbaceous layer

OZ 33: The five main tree species of the 1st tree layer (*bart1bs1 - bart5bs1*):

See definition of 1st tree layer (OZ 24).

Observation area: 10 m radius around the trap site centre.

The name (code) of the most dominant (cover %) up to the fifth most dominant species are noted, according to the species list in Appendix 2.

OZ 34: Cover percentage of the five most important tree species of the 1st tree layer (*deckbart1bs1 - deckbart5bs1*): For the tree species included under OZ 33, the degree of cover is estimated according to 4.

Due to size differences in trees total cover % might exceed 100%.

OZ 35: The five main tree species of the 2nd tree layer (*bart1bs1 - bart5bs1*): See definition of 2st tree layer (OZ 25).

Observation area: 10 m radius around the trap site centre.

The name (code) of the most dominant (cover %) up to the fifth most dominant species are noted. In Appendix 2 all possible species with their code are listed.

OZ 36: Cover percentage of the five most important tree species of the 2^nd^ tree layer (*deckbart1bs2 - deckbart5bs2*): For the tree species listed in OZ 35, cover percentage is estimated according to Table 4. Due to size differences in trees and overlapping layers total cover % might exceed 100%.

OZ 37: The five main tree or shrub species of the shrub layer (*bart1ss - bart5ss*): The shrub layer is formed by all woody plants (trees and shrubs) over 0.5 m in height and a BHD < 7cm.

Observation area: 10 m radius around the trap site centre.

The name (code) of most dominant (cover %) up to the fifth most dominant species are noted. In Appendix 2 all possible species with their name and code are listed.

OZ 38: Cover percentage of the five most important tree or shrub species of the shrub layer (*deckbart1ss - deckbart5ss*): For the species recorded under OZ 37 the cover percentage is estimated according to Table 4. Due to size differences in trees and overlapping layers total cover % might exceed 100%.

OZ 39: The five main tree or shrub species of the herb layer (*bart1ks - bart5ks*): The herb layer is composed of all herbaceous and woody plants up to 0.5m in height. Observation area: 10 m radius around the trap site centre. The name (code) of the most dominant (cover %) up to the fifth most dominant species are noted. In Appendix 2 all possible species with their name and code are listed.

OZ 40: Cover percentage of the five most important tree or shrub species of the herbaceous layer (*deckbart1ss - deckbart5ss*): For the species recorded under OZ 39, the cover percentage is estimated according to Table 4.

## Deadwood

All diameters and heights/lengths for deadwood are estimated only.

OZ 41: Woody litter up to 2 cm (*tlstreu2*): Estimation of the cover percentage of woody litter (small twigs, branches, pieces of bark) up to 2 cm in diameter occurring in the 10 m radius around the trap site centre (Table 4).

OZ 42: Woody litter 2 - 7 cm (*tlstreu7*): Estimation of the cover percentage of woody litter (small twigs, branches, pieces of bark) with a diameter of 2 to 7cm occurring in a 10 m radius around the trap site centre (Table 4).

OZ 43: Coarse woody debris originating from broadleaved trees 7 - 20 cm (*tll7*): All lying deadwood of broadleaved trees between 7 and 20 cm in diameter at the large end found within a 10 m radius of the trap site centre. Cover percentages are estimated according to classes given in Table 4.

OZ 44: Coarse woody debris originating from conifers 7 - 20 cm (*tln7*): All lying deadwood of conifers between 7 and 20 cm in diameter at the large end found within a 10 m radius of the trap site centre. Cover percentages are estimated according to classes given in Table 4.

OZ 45: Coarse woody debris originating from broadleaved trees 20 - 50 cm differentiated in 4 decomposition classes (*tz1ll20 - tz3ll20*): Lying deadwood of broadleaved trees with an diameter at the stronger end of 20 - 50 cm in the respective classes of decomposition according to Table 8.

Observation area: 10 m radius around the trap site centre.

Only absence/presence is assesses, due to the retrospective nature of this study; classification: yes/no.

Table 8. Decomposition classes of deadwood

| ID_TEXT | Decomposition Classes | Description |
| --- | --- | --- |
| 1 | 1-2 | Freshly dead or beginning decomposition |
| 2 | 3 | Advanced decomposition: wood with more or less large "soft rotten" parts, but outlines are still clearly recognizable |
| 3 | 4 | Heavily decomposed, decayed: Outline no longer clearly discernible, lying wood has partially sunken into the ground; standing dead wood is heavily decomposed/rotted. |

OZ 46: Coarse woody debris originating from coniferous trees 20 - 50 cm differentiated in 4 decomposition classes (*tz1ln20 - tz3ln20*): The classification corresponds to that of OZ 46, the only difference being that it applies to coniferous trees.

OZ 47: Coarse woody debris originating from broadleaved trees >50 cm differentiated in 4 decomposition classes (*tz1ll50 - tz3ll50*): Lying deadwood of broadleaved trees with a diameter at the large end of >50 cm in the respective classes of decomposition according to Table 8.

Observation area: 10 m radius around the trap site centre.

Only absence/presence is assesses, due to the retrospective nature of this study; classification: yes/no.

.

OZ 48: Coarse woody debris originating from coniferous trees >50 differentiated in 4 decomposition classes (*tz1ln50 - tz3ln50*):

The classification corresponds to that of OZ 47.

OZ 49: Broadleaved stumps (*stublh*): Number of broadleaved stumps (10m radius).

OZ 50: Conifer stumps (*stubnh*): Number of broadleaved stumps (10m radius).

OZ 51: Snags <7 cm (*tsk7*): Standing deadwood with a BHD < 7 cm regardless of decomposition class.

Observation area: 10 m radius around the trap site centre.

The classification is made according to Table 9.

OZ 52: Broadleaved snags 7-20 cm (*tsl7*): Standing broadleaved deadwood with a BHD of 7-20 cm regardless of decomposition class.

Observation area: 10 m radius around the trap site centre.

Only absence/presence is assesses, due to the retrospective nature of this study; classification: yes/no.

OZ 53: Coniferous snags 7-20 cm (*tsn7*): Standing coniferous deadwood with a BHD of 7-20 cm regardless of the decomposition class.

Observation area: 10 m radius around the trap site centre.

Only the presence or absence is noted. Classification: yes/no.

OZ 54: Broadleaved snags 20-50 cm differentiated by decomposition classes (*tz1sl20 - tz3sl20*): Standing broadleaved deadwood with a BHD of 20-50 cm in the respective decomposition class (Table 12).

Observation area: 10 m radius around the trap site centre.

Only the presence or absence is noted. Classification: yes/no.

OZ 55: Coniferous snags 20-50 cm differentiated in 4 decomposition classes (*tz1sn20 - tz3sn20*): Standing coniferous deadwood with an estimated BHD of 20-50 cm in the respective decomposition class (Table 12).

Observation area: 10 m radius around the trap site centre.

Only the presence or absence is noted. Classification: yes/no

OZ 56: Broadleaved snags >50 cm differentiated in 4 decomposition classes (*tz1sl50 - tz3sl50*): Standing broadleaved deadwood with a BHD of >50 cm in the respective decomposition class (Table 12).

Observation area: 10 m radius around the trap site centre.

Only the presence or absence is noted. Classification: yes/no

OZ 57: Coniferous snags >50 cm differentiated in 4 decomposition classes (*tz1sn20 - tz3sn20*): Standing coniferous deadwood with a BHD >50 cm in the respective decomposition class (Table 12).

Observation area: 10 m radius around the trap site centre.

Only the presence or absence is noted. Classification: yes/no

OZ 58: Root plate (*wteller*): Number of root plates within a 10 m radius of the trap site centre, without recording of tree species or decomposition class.

Observation area: 10 m radius around the trap site centre.

## Linear or small-scale habitats and structures

Red listed habitat types (Finck et al. 2017) and additional structures (habitat area: d ≤ 20 m) that are of special importance to some or all forest dwelling arthropod groups monitored in this program. The habitat list was composed by the Northwest German Forest Research Institute (NW-FVA) and the Senckenberg Society for Nature Research (SGN) based on expert knowledge and literature. Habitat definitions are largely based on the “Red list of endangered biotope types of Germany” (see Finck et al. 2017). The habitat list used here can be found in Appendix 3.

OZ 59: Linear or small-scale habitats (*bio1_10 - bio5_10*): All habitats listed in Appendix 3 must be recorded if present within a 10 m radius of the trap site centre. A maximum of 5 habitat types are documented.

OZ 60: Cover percentage of small-scale biotopes from OZ 59 (*deckbio1_10 - deckbio5_10*): Classification of cover percentages is done according to Table 4.

OZ 61: Small-scale habitats (*bio1_30 - bio5_30*): All habitats listed in Appendix 3 must be recorded if present within the 10 to 30m enclosure ring around the trap site centre. A maximum of 5 habitat types are documented.

OZ 62: Cover percentage of small-scale biotopes from OZ 61 (*deckbio1_30 - deckbio5_30*):

Classification of cover percentages is done according to Table 4.

## Eclector trees

Trees where trunk eclector traps were mounted during field sampling.

OZ 63: Tree species of the eclector tree (*bartekl*): Record the botanical species of the tree to which the eclector was attached according to the species list given in Appendix 2.

OZ 64: BHD of the eclector tree (*dekl*): Diameter (mm) of the tree at 1.3 m height to which the eclector was attached.

OZ 65: Condition Type of eclector tree (*zuekl*): The type of the eclector tree is composed of three classification groups:

1. Life class (LKL) e.g. living = L
2. Position class (PKL) e.g. standing = S
3. Compartment class (CCL): which parts of the tree were still present, e.g. complete tree = v

The determination of the condition type of the eclectic trees is based on Table 13.

Table 9. Chart for determining the condition type of the eclectic trees

| **No.** | **Criteria** | **Class** | | | **Continue to no.** |
| --- | --- | --- | --- | --- | --- |
|  |  | **LKL** | **PKL** | **CCL** |  |
| 1 | Object clearly alive | L |  |  | 3 |
|  | False |  |  |  | 2 |
| 2 | Object clearly dead | T |  |  | 5 |
|  | No clear assignment | A |  |  | 3 |
| 3 | Standing vertically (> 10 grad to the horizontal plane) | L/A | S |  | 4 |
|  | Standing horizontal (≤ 10 grad to the horizontal plane) | L/A | L |  | 4 |
| 4 | No relevant parts of the crown and/or strong branches missing (< 25% of the crown) | L/A | S/L | v | - |
|  | Relevant parts of the crown and/or strong branches missing (≥ 25 % of the crown)  IMPORTANT: this does not include the dieback of understorey trees, these are coded as LSv | L/A | S/L | (v) | - |
|  | Vertical trunk break ≥ 1.3m with living residual stump (vital bark, living buds on branches) | L/A | S | s | - |
|  | Stumps < 1.3m with and without shoots with clearly vital bark | L/A | S | (s) | - |
| 5 | Standing vertically (see 3) | T | S |  | 8 |
|  | Lying (see 4) | T | L |  | 7 |
| 7 | Tree parts (root, trunk, crown) distinguishable | T | L |  | 8 |
|  | Tree parts indistinguishable | T | L | s | - |
| 8 | At least parts of all tree components present (root, trunk, crown (with coarse branches and fine brushwood). | T | S/L | v | - |
|  | At least parts of all tree components present, but fine twigs are missing | T | S/L | (v) |  |
|  | At least one tree part missing |  |  |  | 9 |
| 9 | Trunk (some parts), and crown (pieces), root cut off | T | L | sk | - |
|  | False | T | S/L |  | 10 |
| 10 | Root plate and trunk piece (height of trunk piece ≥ 0.3m), root plate partly or completely lifted out of the ground | T | L | ws | - |
|  | Single tree part | T | S/L | - | 11 |
| 11 | Root plate (height of trunk, if present < 0.3m) | T | S/L | w |  |
|  | No root plate | T | S/L |  | 12 |
| 12 | Trunk piece or strong branches (also the objects originating from the crown area of the stand) | T | S/L |  | 13 |
|  | Crown or crown part (except individual strong branches from the crown area of the stand). | T | S/L | k | - |
| 13 | Stumps ( < 1.3m high) | T | S | (s) | - |
|  | Strong branches, stem parts or stump (splinter pieces stuck in the ground are addressed as lying). | T | S/L | s | - |

OZ 66: Decomposition class of the eclector tree (*zgekl*): Decomposition class of the eclector tree, if it is dead (otherwise not specified). Classes used are the same as for the rest of the deadwood (Table 8).

OZ 67: Eclector tree microhabitats (*hab1ekl - hab5ekl*): A list of (without information on frequency or extent) of habitats occurring at the eclector tree according to Table 10. A maximum of 5 microhabitats can be recorded.

Table 10. Description of microhabitat classes

| Code | Value |
| --- | --- |
| RIND | Bark injury (>10cm): Exposed sapwood, wound is not sealed (10cm in length or width) e.g. felling damage, bark stripping damage, cracks, bark pockets or lightning damage. |
| MUSHROOM | Polypore fruit bodies: perennial, living, dead or highly decomposed fungal fruit bodies of e.g. tinder fungus, red-belted conk, sulphur polypore, Trametes. |
| NEKR | Sap run/necrosis: die back and cracking of bark, sometimes with sap running down the trunk, also necrotic changes and bulging in beech (several exit points must be clearly visible). |
| MOOS | ≥33% coverage of the trunk by mosses and/or foliose and fruticose lichens. For standing trees, the lower 3m of the trunk are addressed. For downed trees, the upper side along the entire length is investigated. |
| HOEHU | Trunk hole below 50cm height: Injuries at the stem base, e.g. skidding damage, cavities due to fungi. |
| HOEHO | Trunk hole above 50cm height and below the crown: trunk injury with cavity formation, e.g. created by branch breakage and rotting, woodpecker holes and other cavities. Cavity can be with or without decomposed organic material, or treehole mould. |

# Literature

Ad-Hoc-AG Boden (2005): [Bodenkundlichen Kartieranleitung](https://de.wikipedia.org/wiki/Bodenkundliche_Kartieranleitung) KA5. Bundesanstalt für Geowissenschaften und Rohstoffe (Ed.). 5. Auflage, Stuttgart, E. Schweitzerbart'sche Verlagsbuchhandlung, Germany. 438 pp.

Arbeitskreis Standortskartierung **(1996): Forstliche Standortsaufnahme: Begriffe, Definitionen, Kennzeichnungen, Erläuterungen; bearb. vom Arbeitskreis Standortskartierung. 7. Auflage., IHW-Verlag, München, Germany. 349 pp.**

Baritz, R. (2003). Humus forms in forests of the northern German lowlands. Stuttgart: Schweizerbart, Techn. Univ. Berlin, Diss.

Bormann, H. (2007). Analysis of the suitability of the german soil texture classification for the regional scale application of physical based hydrological model. *Advances in Geosciences*, *11*, 7-13.

Finck, P, Heinze, S, Raths, U, Riecken, U, Ssymank, A (2017) Rote Liste der gefährdeten Biotoptypen Deutschlands. Bundesamt für Naturschutz (BFN). NaBiV Heft 156. Bonn, Germany. 637 pp.

HMULF (Hessisches Ministerium für Umwelt, Landwirtschaft und Forsten) (2002): Hessische Anweisung für Forsteinrichtungsarbeiten (HAFEA). Wiesbaden, Germany. 72 pp.

# Appendices

## Appendix 1 - Species list

| ID | Common Name | Scientific Name |
| --- | --- | --- |
| 0 | Not recorded | - |
| 10 | Broad-leaved tree | - |
| 50 | Coniferous tree | - |
| 110 | Eiche | Quercus |
| 111 | Stieleiche | Quercus robur |
| 112 | Traubeneiche | Quercus petraea |
| 113 | Roteiche | Quercus rubra |
| 114 | Zerreiche | Quercus cerris |
| 115 | Sumpfeiche | Quercus palustris |
| 211 | Buche | Fagus silvatica |
| 221 | Hainbuche | Carpinus betulus |
| 311 | Esche | Fraxinus excelsior |
| 312 | Blumenesche | Fraxinus ornus |
| 320 | Ahorn | Acer |
| 321 | Bergahorn | Acer pseudoplatanus |
| 322 | Spitzahorn | Acer platanoides |
| 323 | Feldahorn | Acer campestre |
| 324 | Silberahorn | Acer saccharinum |
| 330 | Ulme (Rüster) | Ulmus |
| 331 | Bergulme | Ulmus glabra |
| 332 | Flatterulme | Ulmus laevis |
| 333 | Feldulme | Ulmus minor |
| 340 | Linde | Tilia |
| 341 | Sommerlinde | Tilia platyphyllos |
| 342 | Winterlinde | Tilia cordata |
| 351 | Robinie | Robinia pseudoacacia |
| 352 | Kastanie | Castanea sativa |
| 353 | Nußbaum | Juglans regia |
| 354 | Kirsche | Prunus avium |
| 355 | Wildapfel | Malus silvestris |
| 356 | Wildbirne | Pyrus pyraster |
| 357 | Elsbeere | Sorbus terminalis |
| 358 | Speierling | Sorbus domestica |
| 359 | Mehlbeere | Sorbus intermedia |
| 361 | Tulpenbaum | Liriodendron tulipifera |
| 362 | Hickory | Carya alba |
| 363 | Platane | Platanus acerifolia |
| 364 | Mispel | Mespilus germanica |
| 365 | Wildzwetschge | Prunus spinosa |
| 366 | Stachelbeere | Ribes uva-crispa |
| 371 | Efeu | Hedera helix |
| 372 | Faulbaum | Rhamnus frangula |
| 373 | Frühblühende Traubenkirsche | Prunus padus |
| 374 | Hartriegel | Cornus sanguinea |
| 375 | Hasel | Coryllus avellana |
| 376 | Heckenkirsche | Lonicera xylosteum |
| 377 | Johannisbeere | Ribes spec. |
| 378 | Kornelkirsche | Cornus mas |
| 379 | Kreuzdorn | Rhamnus carthartica |
| 380 | Pfaffenhütchen | Euonymus europaeus |
| 381 | Roter Holunder | Sambucus racemosa |
| 382 | Schneeball | Viburnum lantana/opulus |
| 383 | Schwarzdorn | Prunus spinosa |
| 384 | Schwarzer Holunder | Sambucus nigra |
| 385 | Seidelbast | Daphne mercereum |
| 386 | Stechpalme | Ilex aquifolium |
| 387 | Wacholder | Juniperus communis |
| 388 | Geißblatt, Waldrebe | Clematis vitalba |
| 389 | Weißdorn | Crateagus spec. |
| 390 | Wilde Rose | Rosa canina |
| 391 | Waldrebe | Clematis vitalba |
| 410 | Birke | Betula |
| 411 | Sandbirke | Betula pendula |
| 412 | Moorbirke | Betula pubescens |
| 413 | Japanische Birke | Betula japonica |
| 414 | Hybridbirke | Betula ? |
| 420 | Erle | Alnus |
| 421 | Roterle | Alnus glutinosa |
| 422 | Weißerle | Alnus incana |
| 423 | Grünerle | Alnus viridis |
| 430 | Pappel | Populus spec. |
| 431 | Aspe | Populus tremula |
| 432 | Schwarzpappel | Populus nigra |
| 433 | Graupappel | Populus canescens |
| 434 | Balsampappel | Populus balsamifera |
| 440 | Salweide | Salix caprea |
| 441 | Weide | Salix spec. |
| 442 | Rosskastanie | Aesculus hippocastanum |
| 451 | Eberesche | Sorbus aucuparia |
| 452 | amerik.Traubenkirsche | Prunus serotina |
| 511 | Fichte | Picea abies |
| 512 | Sitkafichte | Picea sitchensis |
| 513 | Omorikafichte(=serb.) | Picea omorika |
| 514 | Stechfichte (=Blau-) | Picea pungens |
| 515 | Schwarzfichte | Picea mariana |
| 516 | Sachalinfichte | Picea glehni |
| 517 | Yedo-Fichte | Picea jezoensis |
| 520 | Tanne | Abies |
| 521 | Weißtanne | Abies alba |
| 522 | Nordmannstanne | Abies nordmanniana |
| 523 | Gr.Küstentanne | Abies grandis |
| 524 | Koloradotanne | Abies concolor |
| 525 | Edeltanne | Abies procera |
| 527 | Sierra-Tanne | Abies concolor var.lowiana |
| 528 | Veitchs-Tanne | Abies veitchii |
| 529 | Purpurtanne | Abies amabilis |
| 531 | Sicheltanne | Cryptomeria japonica |
| 541 | Hemlockstanne | Tsuga heterophylla |
| 542 | Lebensbaum | Thuja plicata |
| 543 | Scheinzypresse | Chamaecyparis |
| 551 | Mammutbaum | Sequoiadendron giganteum |
| 552 | Urweltmammutbaum | Metasequoia glyptostroboides |
| 560 | Eibe | Taxus baccata |
| 562 | Nikko-Tanne | Abies homolepis |
| 563 | Sachalintanne | Abies sachalinensis |
| 564 | Balsamtanne | Abies balsamea |
| 565 | Momi-Tanne | Abies firma |
| 611 | Douglasie | Pseudotsuga menziesii |
| 711 | Gemeine Kiefer | Pinus silvestris |
| 712 | Schwarzkiefer | Pinus nigra |
| 713 | Bergkiefer | Pinus mugo |
| 714 | Bankskiefer | Pinus banksiana |
| 715 | Pechkiefer | Pinus rigida |
| 716 | Drehkiefer | Pinus contorta |
| 731 | Strobe(Weymouthskiefer) | Pinus strobus |
| 741 | Rumelische Kiefer | Pinus peuce |
| 751 | Ki,Herk.Randle Wash. | Pinus ponderosa |
| 761 | Ki,Herk.Oregon | Pinus ponderosa |
| 771 | Ki,Herk.Oregon Deschutes | Pinus ponderosa |
| 810 | Lärche | Larix |
| 811 | Europäische Lärche | Larix decidua |
| 812 | Japan Lärche | Larix kaempferi |
| 813 | Sumpflärche | Larix laricina |
| 814 | Hybridlärche | Larix eurolepis |
| 991 | Besenginster | Cytisus scoparius |
| 999 | Unknown | - |

## Appendix 2 –Habitat types

| **ID** | **Biotop** | **Habitat** |
| --- | --- | --- |
| 1 | Sicker- und Sumpfquellen | Helocrene springs |
| 2 | Sturzquellen | Natural flowing spring |
| 3 | Natürl./naturnah Bachlauf | Natural, near-natural stream |
| 4 | Natürl./naturnah temporäres Fließgewässer. | Natural, near-natural temporary watercourse |
| 5 | Graben, ganzjährig wasserführend | Irrigation ditch, perennial |
| 6 | Graben, temporär/fehlende Wasserführ. | Irrigation ditch, temporarily water-bearing |
| 7 | Natürl./naturnah Stillgewässer | Natural, near-natural ,standing water bodies |
| 8 | Verlandungsbereich Stillgewässer | Aggradation areas of standing water bodies |
| 9 | Tümpel, zeitw. trockenfallend | Pool, temporarily dry |
| 10 | Naturferner Teich | Non-natural Pond |
| 11 | Höhle | Cave |
| 12 | Stollen/Schacht | Shaft, drift |
| 13 | Natürlicher/naturnaher Felsen | Natural, near-natural rock |
| 14 | Solitärer Felsblock, Findling | Boulder, |
| 15 | Natürliche/naturnahe Blockhalde | Natural, near-natural Stone run |
| 16 | Natürliche/naturnahe Schutthalden | Natural, near-natural scree |
| 17 | Steinriegel, Steinhaufen, Steinmauer | Stone bars, pile of stones, stone wall |
| 18 | Steinbruch | Stone quarry |
| 19 | vegetationsfreie (Weg)kante a. Sand | road embankment without vegetation, sand |
| 20 | vegetationsfreie (Weg)kante a. Löss/Lehm | road embankment without vegetation, loess/loam |
| 21 | Vegetationsarme Kies- und Schotterfläche | area dominated by grit or gravel sparsely vegetated |
| 22 | Vegetationsarme Sandflächen | sparsely vegetated areas dominated by sand |
| 23 | Magerrasen | Semi-natural grasslands on nutrient poor soils |
| 24 | Zwergstrauchheide | Dwarf Shrub Heath |
| 25 | Extensiv-Grünland (m.o.w. artenreich, fr | Extensively used grassland, generally species-rich and moist |
| 26 | Extensiv-Grünland (m.o.w. artenreich, na | Extensively used grassland, generally species-rich and wet |
| 27 | Intensivgrünland/Äsungsfläche (artenarm) | Intensively managed grasslands/ grazing areas (species-poor) |
| 28 | Wildacker | wildlife food plot |
| 29 | Niedermoor/Sumpf (Seggenried, Röhricht) | Blanket bogs /swamp (sedge reeds, reedbed) |
| 30 | Hochmoor | Raised bog |
| 31 | unbefestigter Weg/Schneise (grasdominiert) | Unpaved road/trail (dominated by grass) |
| 32 | unbefestigter Weg/Schneise (Offenboden) | Unpaved road/trail (bare soil) |
| 33 | Geschotterter Weg/Platz | Gravel road/ground |
| 34 | Asphaltierter Weg/Straße/Platz | Paved road/streets/open areas |
| 35 | Hohlweg | Sunken road |
| 36 | Wald- und Gehölzsäume/Waldrand | Forest edge |
| 37 | Gebüsch (heim. Arten) | Bushes (native species) |
| 38 | Wallhecke, Knick | Hedge bank |
| 39 | Kahlschlag/Lichtungsflur (krautige Veg.) | Clear cut/ Clearing (herbaceous plants) |
| 40 | Neophyten-Staudenfluren | Neophytes forb stands |
| 41 | Adlerfarn-Dominanzbestand, gehölzfrei | Braken dominated stands, without woody plants |
| 42 | Reitgras-Dominanzbestand, gehölzfrei | Reedgras dominated stands, without woody plants |
| 43 | Himbeer-/Brombeergestrüpp | Rasberry/ blackberry shrubs |
| 44 | Kirrung | Wildlife bait site |
| 45 | Insektennest | Insect nest |
| 46 | Kleinsäugerbau | Small mammal burrow |
| 47 | Großsäugerbau | Great mammal burrow |
